# Deciphering the genetic architecture and ethnographic distribution of IRD in three ethnic populations by whole genome sequence analysis

**DOI:** 10.1371/journal.pgen.1009848

**Published:** 2021-10-18

**Authors:** Pooja Biswas, Adda L. Villanueva, Angel Soto-Hermida, Jacque L. Duncan, Hiroko Matsui, Shyamanga Borooah, Berzhan Kurmanov, Gabriele Richard, Shahid Y. Khan, Kari Branham, Bonnie Huang, John Suk, Benjamin Bakall, Jeffrey L. Goldberg, Luis Gabriel, Naheed W. Khan, Pongali B. Raghavendra, Jason Zhou, Sindhu Devalaraja, Andrew Huynh, Akhila Alapati, Qais Zawaydeh, Richard G. Weleber, John R. Heckenlively, J. Fielding Hejtmancik, Sheikh Riazuddin, Paul A. Sieving, S. Amer Riazuddin, Kelly A. Frazer, Radha Ayyagari

**Affiliations:** 1 Shiley Eye Institute, University of California San Diego, La Jolla, California, United States of America; 2 School of Biotechnology, REVA University, Bengaluru, Karnataka, India; 3 Retina and Genomics Institute, Yucatán, México; 4 Laboratoire de Diagnostic Moleculaire, Hôpital Maisonneuve Rosemont, Montreal, Quebec, Canada; 5 Ophthalmology, University of California San Francisco, San Francisco, California, United States of America; 6 Institute for Genomic Medicine, University of California, San Diego, La Jolla, California, United States of America; 7 GeneDx, Gaithersburg, Maryland, United States of America; 8 The Wilmer Eye Institute, Johns Hopkins University School of Medicine, Baltimore, Maryland, United States of America; 9 Ophthalmology & Visual Science, University of Michigan Kellogg Eye Center, Ann Arbor, Michigan, United States of America; 10 Ophthalmology, University of Arizona College of Medicine Phoenix, Phoenix, Arizona, United States of America; 11 Byers Eye Institute, Stanford, Palo Alto, California, United States of America; 12 Genetics and Ophthalmology, Genelabor, Goiânia, Brazil; 13 School of Regenerative Medicine, Manipal University, Bengaluru, Karnataka, India; 14 Casey Eye Institute, Oregon Health & Science University, Portland, Oregon, United States of America; 15 Ophthalmic Genetics and Visual Function Branch, National Eye Institute, National Institutes of Health, Bethesda, Maryland, United States of America; 16 National Centre of Excellence in Molecular Biology, University of the Punjab, Lahore, Pakistan; 17 Allama Iqbal Medical College, University of Health Sciences, Lahore, Pakistan; 18 National Eye Institute, Bethesda, Maryland, United States of America; 19 Ophthalmology & Vision Science, UC Davis Medical Center, California, United States of America; 20 Department of Pediatrics, Rady Children’s Hospital, Division of Genome Information Sciences, San Diego, California, United States of America; Case Western Reserve University, UNITED STATES

## Abstract

Patients with inherited retinal dystrophies (IRDs) were recruited from two understudied populations: Mexico and Pakistan as well as a third well-studied population of European Americans to define the genetic architecture of IRD by performing whole-genome sequencing (WGS). Whole-genome analysis was performed on 409 individuals from 108 unrelated pedigrees with IRDs. All patients underwent an ophthalmic evaluation to establish the retinal phenotype. Although the 108 pedigrees in this study had previously been examined for mutations in known IRD genes using a wide range of methodologies including targeted gene(s) or mutation(s) screening, linkage analysis and exome sequencing, the gene mutations responsible for IRD in these 108 pedigrees were not determined. WGS was performed on these pedigrees using Illumina X10 at a minimum of 30X depth. The sequence reads were mapped against hg19 followed by variant calling using GATK. The genome variants were annotated using SnpEff, PolyPhen2, and CADD score; the structural variants (SVs) were called using GenomeSTRiP and LUMPY. We identified potential causative sequence alterations in 61 pedigrees (57%), including 39 novel and 54 reported variants in IRD genes. For 57 of these pedigrees the observed genotype was consistent with the initial clinical diagnosis, the remaining 4 had the clinical diagnosis reclassified based on our findings. In seven pedigrees (12%) we observed atypical causal variants, i.e. unexpected genotype(s), including 4 pedigrees with causal variants in more than one IRD gene within all affected family members, one pedigree with intrafamilial genetic heterogeneity (different affected family members carrying causal variants in different IRD genes), one pedigree carrying a dominant causative variant present in pseudo-recessive form due to consanguinity and one pedigree with a de-novo variant in the affected family member. Combined atypical and large structural variants contributed to about 20% of cases. Among the novel mutations, 75% were detected in Mexican and 50% found in European American pedigrees and have not been reported in any other population while only 20% were detected in Pakistani pedigrees and were not previously reported. The remaining novel IRD causative variants were listed in gnomAD but were found to be very rare and population specific. Mutations in known IRD associated genes contributed to pathology in 63% Mexican, 60% Pakistani and 45% European American pedigrees analyzed. Overall, contribution of known IRD gene variants to disease pathology in these three populations was similar to that observed in other populations worldwide. This study revealed a spectrum of mutations contributing to IRD in three populations, identified a large proportion of novel potentially causative variants that are specific to the corresponding population or not reported in gnomAD and shed light on the genetic architecture of IRD in these diverse global populations.

## Author summary

The study was performed to identify the underlying cause of inherited retinal degeneration (IRD) in 409 individuals from 108 families. Primarily, these families were recruited from three different geographic regions: Mexico, Pakistan and European Americans from the United States. Blood samples were collected from all individuals for genome analysis. This analysis detected causative variants in 61 out of the 108 pedigrees. A total of 93 gene variants were found in the 61 families. Among these, 54 were previously reported as causative variants and the remaining 39 have not been reported in IRD pedigrees. Interestingly, 54% of these novel variants were not listed in gnomAD. In addition to these findings, complex causative genotypes were observed in 20% of pedigrees. Overall, causative variants were detected in 63% Mexican, 60% Pakistani and 45% European American pedigrees. This study revealed the distribution of IRD causative variants in pedigrees with diverse ethnic and geographic backgrounds.

## Introduction

Inherited retinal degenerations (IRDs) are a group of diseases, which result in dysfunction or progressive degeneration of retinal cells causing a profound bilateral loss of vision. IRDs are relatively rare. It is currently estimated that IRDs affect 1 in 3000 individuals [1]. Significant heterogeneity has been reported in the phenotype of IRD patients with a wide variation in the age of onset, rate of progression, severity of the disease, and clinical symptoms. Variants in the same gene may also lead to marked diverse phenotypes as well as result in different patterns of inheritance. Currently, at least 271 genes are known to be associated with IRD [2].

Retinal disease genes have been identified previously by linkage analysis, homozygosity mapping, and sequencing the coding regions of several genes associated with genetic and genomic markers. The subsequent development of targeted screening panels for pathogenic variants in known IRD genes greatly improved genetic diagnosis but failed to identify novel variants and novel genes involved in IRD [3–5]. Gene arrays to selectively capture and sequence candidate genes are reported to result in the identification of mutations in 60%-70% of IRD patients [3, 5, 6]. Advances in whole-exome sequencing (WES) enabled the identification of causal variants associated with Mendelian diseases in known or novel genes efficiently [7, 8]. Nevertheless, about 30%-40% of cases remain unresolved. Further, while the majority of studies conducted so far focused on selected populations, the genomic architecture of IRD in certain populations remains unknown.

The affordable cost structure of whole-genome sequencing in recent years [9–13] has enabled the analysis of all genes including their untranslated regions and provided opportunities to identify causal variants in patients with IRDs with broad genetic and phenotypic heterogeneity. Utilizing these advances in the current study, we present the genetic analysis of IRD in 108 pedigrees. These pedigrees are mainly from three populations: the understudied populations from Pakistan (Punjab province) and Mexico as well as the well-studied European American population (individuals of European ancestry from North America). Analysis of these pedigrees revealed atypical sequence alterations and provided a glimpse of the genetic architecture of IRD in these distinctly diverse global populations.

## Results

### Pedigrees analyzed

Whole-genome sequence data were obtained on 404 subjects from 108 unrelated pedigrees with a diagnosis of inherited retinal dystrophy. The study cohort included pedigrees from Mexico (35), Pakistan (15), Ashkenazi Jewish (2), India (2), and USA (European ancestry) (54).

The pattern of inheritance was observed to be recessive in 76 pedigrees, dominant in 25, and X-linked in 7. However, after completing the analysis, the pattern of inheritance was corrected in 4 pedigrees based on the causative mutations detected. One pedigree with multiple consanguineous marriages (RF.197.0113) was originally classified as recessive but determined to be dominant with a pseudo-recessive pattern of inheritance. Similarly, two pedigrees RF.VI123.0514 and RF.VI153.0216 were originally classified as dominant and recessive respectively but mutations in X-linked genes were identified as the underlying cause of the phenotype. One pedigree originally classified as dominant (RF.VI116.1215) was re-classified as recessive.

### WGS sequence analysis

Analysis of sequence data identified 202 female and 202 male subjects consistent with our records and validated relationships based on identity by descent (IBD) mapping analysis. The total number of reads obtained on each individual ranged from 765 million to 1,903 million, of which 78% ~ 95% were detected as appropriately mapped reads indicating the high quality of sequence data. Analysis using GATK best practice pipeline identified 30,071,475 single nucleotide variants (SNVs) in total, including 23,409,845 single nucleotide polymorphisms (SNPs) and 6,661,630 INDELs. The number of variants in each sample ranged from 3.77 to 4.84 million SNVs. A total of 18,301,653 known and 11,769,822 novel (based on dbSNP147) SNVs were observed in 404 subjects. Among the total number of identified SNVs, 21,026,019 (70%) were identified as very rare SNVs (allele frequency < 0.001). The rare and moderate/possibly disease-causing SNVs included 186,501 (0.61%) while only 53,101 (0.18%) of them were predicted to be deleterious/probably damaging.

#### (i) Small variants (SNVs and small INDELs)

3.77 to 4.84 million SNVs including ~850,000 small INDELs were detected from autosomes in every individual and no outliers or plate biases were observed. Similarly, no outliers were observed in the X and Y chromosome data. The heterozygous and homozygous ratios were normal on autosomes as well as sex chromosomes in each female and male sample. Among the total SNVs observed, 112,335 (0.37%) were annotated as missense variants. These include 79,428 (71%) known and 32,907 (29%) novel variants.

#### (ii) Copy Number Variants (CNVs)

We observed a total of 56,299 CNVs including 25,357 deletions, 13,223 duplications, and 17,719 insertions in 404 samples. More than half of the CNVs, 29,142 (52%) were found to be common as they were found in more than 30 samples. The CNV calling software (GenomeStrip) detected CNVs with lengths greater than 1000bp. In our analysis, we identified CNVs ranging from 1000bp to 313,600bp. The CNVs were called with a quality score, and those <1 were classified as likely false positives.

#### (iii) ExAC Z score distribution in Retina genes

ExAC database has constraint Z scores for 18,225 genes. In our analysis, we included 271 retinal disease-associated genes from the RetNet database [2] and 58 other possible candidate genes associated with IRD based on their expression in relevant cells and function. Among these were 311 genes listed in the ExAC database including 183 recessive, 75 dominant, 9 X-linked genes, and 44 undefined genes. Positive Z scores indicated increased variation intolerance and therefore these 311 genes had fewer variants than expected. Autosomal dominant/X-linked IRD related genes were highly conserved and sequence alterations in these genes have among the highest Z-scores. Therefore, we used Z-scores to prioritize the candidate variants for dominant and X-linked related genes but not for recessive genes.

### Causative variants detected in IRD associated genes

Analysis of WGS variants identified 93 causative variants (88 SNVs and 5 CNVs) in 61 of the pedigrees (57%). 89 of these causative variants include 35 novel and 54 previously reported variants detected in 45 known IRD genes in 59 pedigrees (Tables 1, 2, 3, 4 and S1). In two additional pedigrees, 4 novel mutations were identified in 2 newly classified novel IRD associated genes, *AGBL5* and *IFT88* [10, 14].

#### (i) Novel potentially causative variants detected in known IRD genes

**Fig 1:**
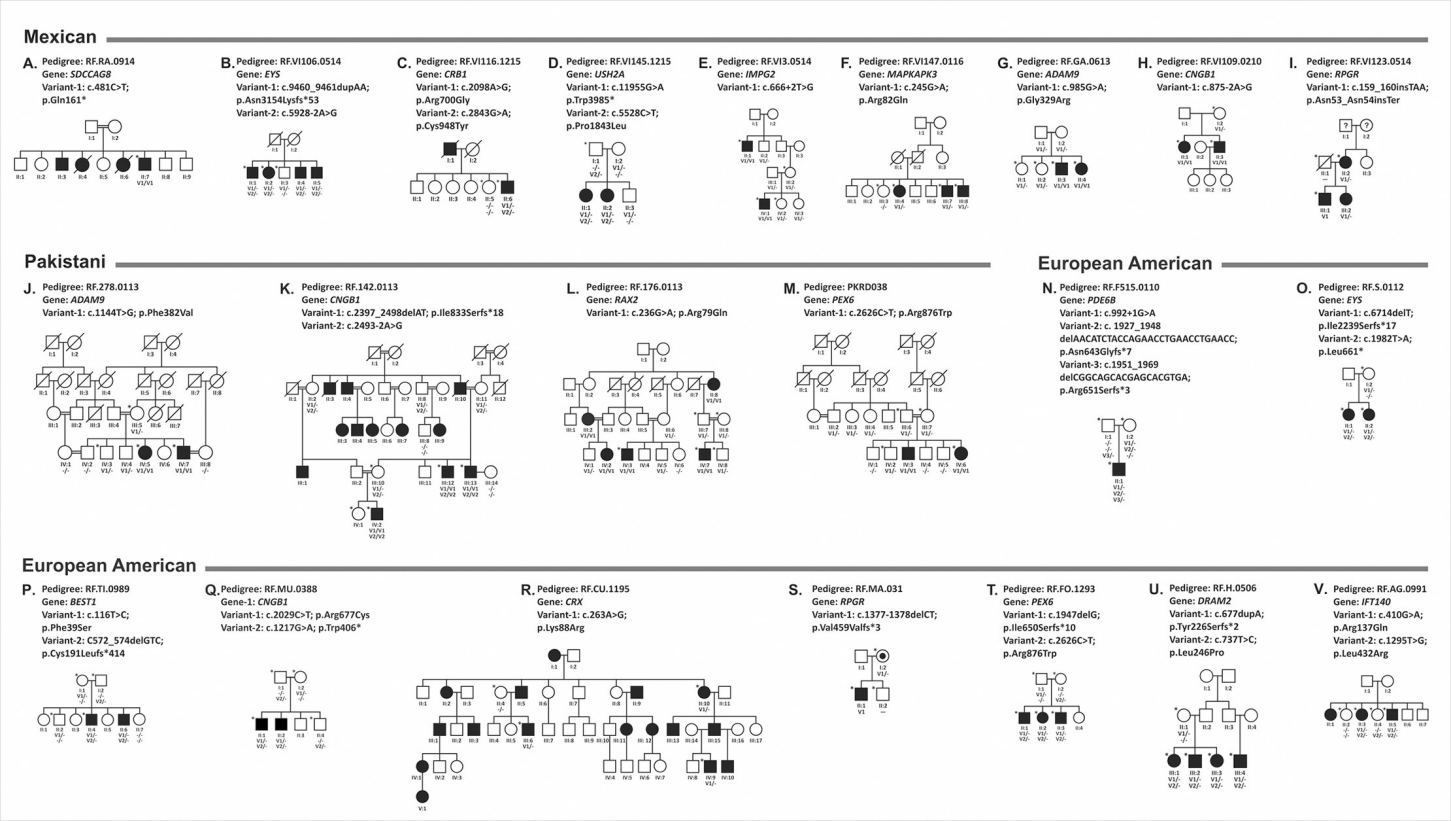
Pedigrees with novel mutations. The segregation analysis of 22 pedigrees showed 26 rare potentially pathogenic novel variants along with 8 previously reported mutations in 17 IRD associated genes. These include 9 homozygous, 21 compound heterozygous, two dominant acting heterozygous, and two X-linked variants. Pedigrees A-I are Mexican, J-M are Pakistani and N-V are European American. The asterisk indicates the availability of whole-genome sequencing data.

In 22 pedigrees (9 Mexican, 4 Pakistani and 9 European American), 26 rare, potentially pathogenic novel (not previously reported as causative) variants in 17 different known IRD genes were identified as likely causative mutations. Seven of these pedigrees also have 8 previously reported mutations in known IRD genes. Among the variants detected, 9 were homozygous (in 8 pedigrees), 21 compound heterozygous (in 10 pedigrees), 2 dominant acting heterozygous (in 2 pedigrees), and 2 were X-linked variants (in 2 pedigrees) (Table 1). Five of these variants were nonsense, 15 missense, 9 frameshift, and 5 intronic splice altering variants. Sanger sequencing analysis of all available family members confirmed co-segregation of candidate variants with IRD (Fig 1).

**Table 1:** Pedigrees with novel potentially causative variants in known IRD genes.

Pedigree	Ethnicity	Chr	Position	Gene (NM_ID)	Zygosity in affected individual #	rsID	gnomAD	cDNA	Protein change	Prediction by PolyPhen2	Population allele frequency (AF) (No of Homozygotes)	References
(A) RF.RA.0914	Mexican	Chr 1	243286332	*SDCCAG8 (NM_006642*.*5)*	Homozygous	rs797045947	0.00000398	c.481C>T	p.Gln161*	N/A	Latino (0.00002892) (0)	This study
(B) RF.VI106.0514	Mexican	Chr 6	64430528	*EYS (NM_001142800*.*2)*	Comp Het	NL	NL	c.9460_9461dupAA	p.Asn3154Lysfs*53	N/A	NL	This study
			65098735			rs181169439	0.0000263	c.5928-2A>G	N/A	N/A	Latino (0.0001412), European (0.00002139) (0)	(15)
(C) RF.VI116.1215	Mexican	Chr 1	197391056	*CRB1 (NM_201253*.*3)*	Comp Het	rs931715897	0.00000401	c.2098A>G	p.Arg700Gly	probably damaging	Latino (0.00002894) (0)	This study
			197403836			rs62645748	0.0002027	c.2843G>A	p.Cys948Tyr	probably damaging	European (0.0003895), Latino (0.0001133) (0)	(16)
(D) RF.VI145.1215	Mexican	Chr 1	215901483	*USH2A (NM_206933*.*4)*	Comp Het	NL	NL	c.11955G>A	p.Trp3985*	N/A	NL	This study
			216251475			rs200209833	0.000003989	c.5528C>T	p.Pro1843Leu	probably damaging	East Asian (0.00005444) (0)	(17)
(E) RF.VI3.0514	Mexican	Chr 3	100994505	*IMPG2 (NM_016247*.*4)*	Homozygous	NL	NL	c.666+2T>G	N/A	N/A	NL	This study
(F) RF.VI147.0116	Mexican	Chr 3	50677822	*MAPKAPK3 (NM_001243925*.*2)*	Heterozygous	rs777383707	0.00001593	c.245G>A	p.Arg82Gln	probably damaging	Latino (0.00008680), South Asian (0.00003268) (0)	This study
(G) RF.GA.0613	Mexican	Chr 8	38883392	*ADAM9 (NM_003816*.*3)*	Homozygous	NL	NL	c.985G>A	p.Gly329Arg	probably damaging	NL	This study
(H) RF.VI109.0210	Mexican	Chr 16	57984446	*CNGB1 (NM_001297*.*5)*	Homozygous	NL	NL	c.875-2A>G	N/A	N/A	NL	This study
(I) RF.VI123.0514	Mexican	Chr X	38182193	*RPGR (NM_000328*.*3)*	Hemizygous	NL	NL	c.159_160insTAA	p.Asn54*	N/A	NL	This study
(J) RF.278.0113	Pakistani	Chr 8	38899478	*ADAM9 (NM_003816*.*3)*	Homozygous	NL	NL	c.1144T>G	p.Phe382Val	probably damaging	NL	This study
(K) RF.142.0113	Pakistani	Chr 16	57938773	*CNGB1 (NM_001297*.*5)*	Homozygous	rs1390543436	0.00002004	c.2497_2498delAT	p.Ile833Serfs*18	N/A	South Asian (0.0001634) (0)	This study
			57938781		Homozygous	rs745319716	0.00002005	c.2493-2A>G	N/A	N/A	South Asian (0.0001634) (0)	(18)
(L) RF.176.0113	Pakistani	Chr 19	3770938	*RAX2 (NM_001319074*.*4)*	Homozygous	rs779301810	0.00002017	c.374G>A	p.Arg79Gln	probably damaging	South Asian (0.0001303), East Asian (0.00008864) (0)	This study
(M) PKRD038	Pakistani	Chr 6	42932853	*PEX6 (NM_000287*.*4)*	Homozygous	rs267608246	0.000003977	c.2626C>T	p.Arg876Trp	probably damaging	European (0.000008793) (0)	This study
(N) RF.F515.0110	European American	Chr 4	648678	*PDE6B (NM_000283*.*4)*	Comp Het	rs898144119	0.000007962	c.992+1G>A	N/A	N/A	South Asian (0.00006533) (0)	This study
			657564			rs1296042817	0.00000713	c.1927_1948delAA CATCTACCAGA ACCTGAACC	p.Asn643Glyfs*7	N/A	European (0.000007841) (0)	(19)
			657588			rs1239431411	0.00001068	c.1951_1969delCG GCAGCACGAGC ACGTGA	p.Arg651Serfs*3	N/A	Ashkenazi Jewish (0.00009705), European (0.000007825) (0)	This study
(O) RF.S.0112	European American	Chr 6	64776241	*EYS (NM_001142800*.*2)*	Comp Het	rs914795218	NL	c.6714delT	p.Ile2239Serfs*17	N/A	European (0.00006506), Latino (0.00004083) (0)	(19)
			66005797			NL	NL	c.1982T>A	p.Leu661*	N/A	NL	This study
(P) RF.TI.0989	European American	Chr 11	61719394	*BEST1 (NM_004183*.*4)*	Comp Het	NL	NL	c.116T>C	p.Phe39Ser	probably damaging	NL	This study
			61725654			NL	NL	c.572_574delGTC	p.Cys191Leufs*414	N/A	NL	This study
(Q) RF.MU.0388	European American	Chr 16	57951309	*CNGB1 (NM_001297*.*5)*	Comp Het	NL	0.00009912	c.2029C>T	p.Arg677Cys	probably damaging	African, South Asian, European	(20)
			57973489			NL	0.000008269	c.1217G>A	p.Trp406*	N/A	African (0.0002480)	This study
(R) RF.CU.1195	European American	Chr 19	48342587	*CRX (NM_000554*.*6)*	Heterozygous	NL	NL	c.263A>G	p.Lys88Arg	possibly damaging	NL	This study
(S) RF.MA.031	European American	Chr X	38156572	*RPGR (NM_000328*.*3)*	Hemizygous	rs62653029	NL	c.1377_1378delCT	P.Val459Valfs*3	N/A	NL	This study
(T) RF.FO.1293	European American	Chr 6	42932993	*PEX6 (NM_000287*.*4)*	Comp Het	rs764227040	0.00001061	c.2585G>T	p.Gly862Val	probably damaging	European (0.00002324) (0)	This study
			42934533			rs267608227	0.00002122	c.1947delG	p.Ile650Serfs*10	N/A	African (0.00004008),	(18)
(U) RF.H.0506	European American	Chr 1	111660846	*DRAM2 (NM_001349884*.*2)*	Comp Het	rs148031211	0.0003706	c.737T>C	p.Leu246Pro	probably damaging	European (0.0006604),	This study
			111661442			rs376487338	0.00002472	c.677dupA	p.Tyr226Serfs*2	N/A	European (0.00005570) (0)	This study
(V) RF.AG.0991	European American	Chr 16	1642549	*IFT140 (NM_014714*.*4)*	Comp Het	rs145718272	0.0002315	c.410G>A	p.Arg137Gln	probably damaging	Ashkenazi Jewish (0.002221), European (0.0002790), Latino (0.0002259) (0)	This study
			1634282			NL	NL	c.1295T>G	p.Leu432Arg	possibly damaging	NL	This study

Note: NL: Not listed; N/A: Not applicable, AF: Allele frequency. Zygosity in affected individual^#^: The causative variants detected in Pedigrees C to V co-segregated with disease (Table 1 and Fig 1). The parental DNA of pedigrees, RF.RA.0914 (Fig 1A) and RF.VI106.0514 (Fig 1B) are not available for segregation analysis.

*Pedigrees with variants of uncertain significance (VUS)*. Among the novel potentially causative variants observed in Table 1 and Fig 1, five variants found in *RAX2 (p*.*Arg79Gln in RF*.*176*.*0113)*, *PEX6 (p*.*Arg876Trp in PKRD038 and p*.*Gly862Val in RF*.*FO*.*1293)*, *DRAM2* (p.Leu246Pro in RF.H.0506) and *IFT140* (p.Arg137Gln in RF.AG.0991) are listed in CinVar as VUS.

Further analysis of the IRD pedigrees with these five VUS did not detect additional potentially causative variants in known or novel genes that are sufficient to cause disease. Future experimental evaluation of novel potentially pathogenic causative variants and VUS, and detection of these variants in additional unrelated IRD cases will provide evidence for an appropriate classification of their clinical relevance.

#### (ii) Previously reported mutations detected in known IRD genes

**Fig 2:**
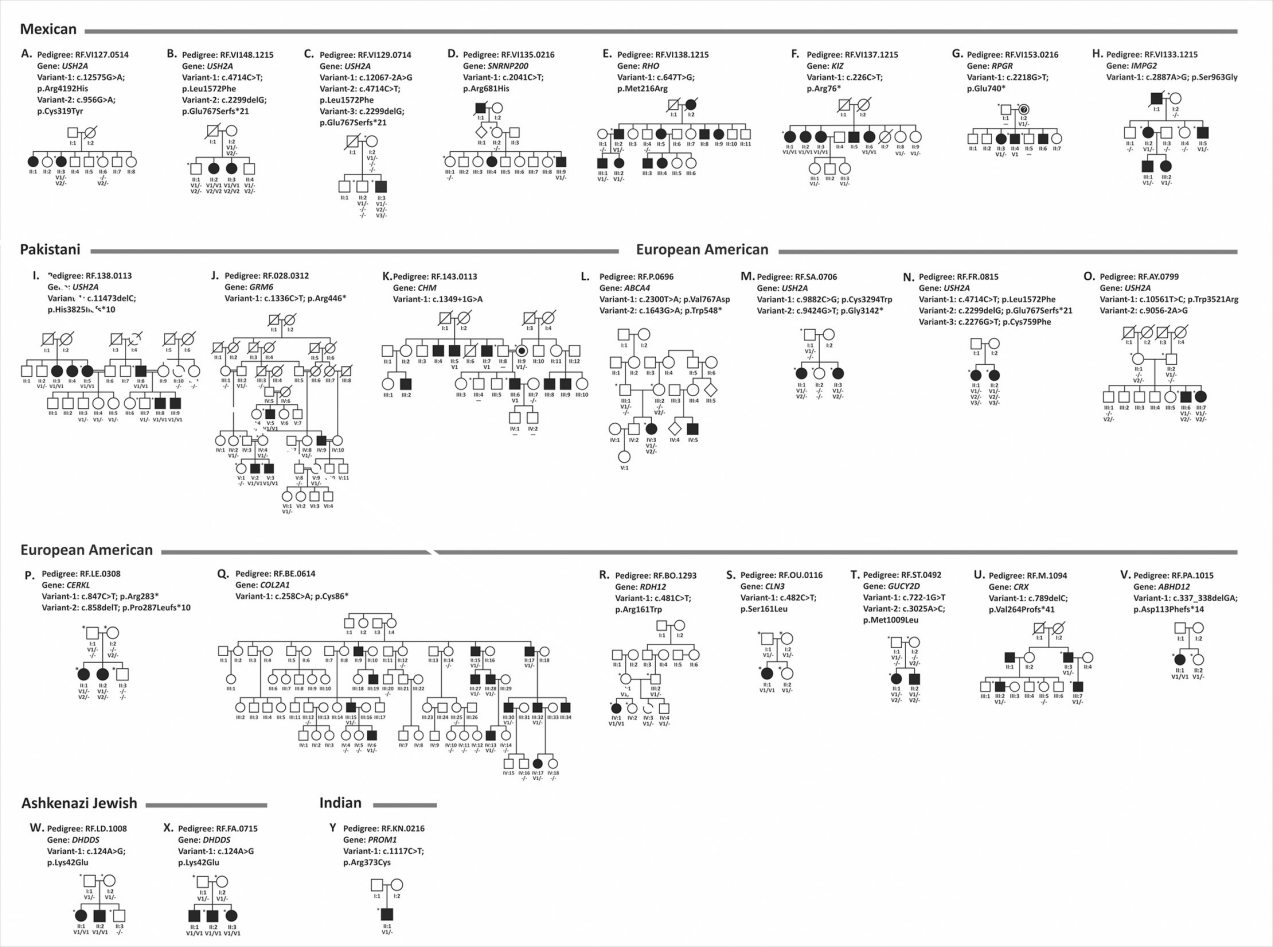
Pedigrees with previously reported mutations. The segregation analysis revealed 35 previously reported mutations that were identified in 25 pedigrees. There were seven frameshift, 17 missense, seven premature stop codon mutations, and four splice site altering changes. Of these ten are homozygous, six dominant heterozygous, two X-linked, and 17 compound heterozygous mutations found in these pedigrees. Pedigrees A-H are Mexican, I-K are Pakistani and L-V are European American, W-X are Ashkenazi Jewish and Y is Indian. The asterisk indicates the availability of whole-genome sequencing data.

Thirty-five previously reported mutations in 18 known IRD genes were identified in 25 pedigrees (Table 2 and Fig 2). These pedigrees include 8 Mexican, 3 Pakistani, 14 European American including two of Ashkenazi Jewish origin and one Indian. Seven frameshift, 17 missense, 7 premature stop codon mutations, and 4 splice site altering changes were observed. Ten homozygous (in 9 pedigrees), 6 dominantly acting heterozygous (in 6 pedigrees), 2 X-linked (in 2 pedigrees) and 17 compound heterozygous mutations (in 8 pedigrees) were found in these 25 pedigrees (Table 2).

**Table 2:** Pedigrees with previously reported mutations in IRD genes.

Pedigree	Ethnicity	Chr	Position	Gene (NM_ID)	Zygosity in affected individuals	rsID	gnomAD AF	cDNA	Protein change	Prediction by PolyPhen2	Variant listed in population	Reference
(A) RF.VI127.0514	Mexican	Chr 1	215848678	*USH2A (NM_206933*.*4)*	Comp Het	rs199605265	0.0005661	c.12575G>A	p.Arg4192His	benign	Ashkenazi Jewish, Latino, European	(21)
			216498834			rs121912599	0.00005979	c.956G>A	p.Cys319Tyr	probably damaging	Latino	(22)
(B) RF.VI148.1215	Mexican	Chr 1	216270469	*USH2A (NM_206933*.*4)*	Homozygous	rs111033333	0.0007287	c.4714C>T	p.Leu1572Phe	probably damaging	Latino, European, African	(23, 24)
			216420436			rs80338903	0.0007017	c.2299delG	p.Glu767Serfs*21	N/A	Latino, European, African	(25)
(C) RF.VI129.0714	Mexican	Chr 1	215853720	*USH2A (NM_206933*.*4)*	Comp Het	rs397517978	0.00008414	c.12067-2A>G	N/A	N/A	Latino, European	(26)
			216420436			rs80338903	0.0007017	c.2299delG	p.Glu767Serfs*21	N/A	Latino, European, African	(25)
(D) RF.VI135.0216	Mexican	Chr 2	96958829	*SNRNP200 (NM_014014*.*5)*	Heterozygous	NL	NL	c.2041C>T	p.Arg681His	probably damaging	NL	(27)
(E) RF.VI138.1215	Mexican	Chr 3	129251210	*RHO (NM_000539*.*3)*	Heterozygous	NL	NL	c.647T>G	p.Met216Arg	benign	NL	(28, 29)
(F) RF.VI137.1215	Mexican	Chr 20	21117104	*KIZ (NM_018474*.*6)*	Homozygous	rs202210819	0.0003897	c.226C>T	p.Arg76*	N/A	NL	(30)
(G) RF.VI153.0216	Mexican	Chr X	38146034	*RPGR (NM_000328*.*3)*	Hemizygous	NL	NL	c.2218G>T	p.Glu740*	N/A	NL	(31)
(H) RF.VI133.1215	Mexican	Chr 3	100961667	*IMPG2 (NM_016247*.*4)*	Heterozygous	NL	NL	c.2887A>G	p.Ser963Gly	probably damaging	NL	(32)
(I) RF.138.0113	Pakistani	Chr 1	215916593	*USH2A (NM_206933*.*4)*	Homozygous	rs774677256	0.000008	c.11473delC	p.His3825Ilefs*10	N/A	South Asian	(7)
(J) RF.028.0312	Pakistani	Chr 5	178415954	*GRM6 (NM_000843*.*4)*	Homozygous	rs764476239	0.00001609	c.1336C>T	p.Arg446*	N/A	South Asian	(33, 34)
(K) RF.143.0113	Pakistani	Chr X	85901083	*CHM (NM_000390*.*4)*	Hemizygous	NL	NL	c.1349+1G>A	N/A	N/A	NL	(35)
(L) RF.P.0696	European American	Chr 1	94522239	*ABCA4 (NM_000350*.*3)*	Comp Het	rs61751395	0.00001991	c.2300T>A	p.Val767Asp	benign	European	(36)
			94528785			NL	NL	c.1643G>A	p.Trp548*	N/A	NL	(37)
(M) RF.SA.0706	European American	Chr 1	215972325	*USH2A (NM_206933*.*4)*	Comp Het	rs749228276	0.00001647	c.9882C>G	p.Cys3294Trp	probably damaging	European	(8)
			215990485			rs397518048	0.00002398	c.9424G>T	p.Gly3142*	N/A	European	(8)
(N) RF.FR.0815	European American	Chr 1	216420460	*USH2A (NM_206933*.*4)*	Comp Het	rs80338902	0.0007825	c.2276G>T	p.Cys759Phe	probably damaging	Latino, European, African	(38)
			216420436			rs80338903	0.0007017	c.2299delG	p.Glu767Serfs*21	N/A	Latino, European, African	(25)
			216270469			rs111033333	0.0007287	c.4714C>T	p.Leu1572Phe	probably damaging	Latino, European, African	(39)
(O) RF.AY.0799	European American	Chr 1	215956104	*USH2A (NM_206933*.*4)*	Comp Het	rs111033264	0.00001647	c.10561T>C	p.Trp3521Arg	probably damaging	European, Latino	(23, 40)
			216017840			rs754970095	0.00001233	c.9056-2A>G	N/A	N/A	European	(38)
(P) RF.LE.0308	European American	Chr 2	182423332	*CERKL (NM_201548*.*5)*	Comp Het	rs398122964	0.00002009	c.858delT	p.Pro287Leufs*10	N/A	African, European	(41)
			182423344			rs121909398	0.000397	c.847C>T	p.Arg283*	N/A	European, South Asian	(42)
(Q) RF.BE.0614	European American	Chr 12	48393736	*COL2A1 (NM_001844*.*5)*	Heterozygous	rs794727261	NL	c.258C>A	p.Cys86*	N/A	NL	(43)
(R) RF.BO.1293	European American	Chr 14	68193730	*RDH12 (NM_152443*.*3)*	Homozygous	rs759408031	0.00002475	c.481C>T	p.Arg161Trp	probably damaging	European, South Asian	(44)
(S) RF.OU.0116	European American	Chr 16	28497950	*CLN3 (NM_001042432*.*2)*	Homozygous	NL	NL	c.482C>T	p.Ser161Leu	probably damaging	NL	(45)
(T) RF.ST.0492	European American	Chr 17	7907169	*GUCY2D (NM_000180*.*4)*	Comp Het	rs773327031	0.000004048	c.722-1G>T	N/A	N/A	African	(46)
			7919141			rs61750188	0.00000812	c.3025A>C	p.Met1009Leu	probably damaging	European	(47)
(U) RF.M.1094	European American	Chr 19	48343109	*CRX (NM_000554*.*6)*	Heterozygous	NL	NL	c.789delC	p.Pro263Profs*41 p.Val264Trpfs*10	N/A	NL	(48, 49)
(V) RF.PA.1015	European American	Chr 20	25304044	*ABHD12 (NM_001042472*.*3)*	Homozygous	NL	NL	c.337_338delGA	p.Asp113Phefs*14	N/A	NL	(50)
(W) RF.LD.1008	Ashkenazi Jewish	Chr 1	26764719	*DHDDS (NM_205861*.*3)*	Homozygous	rs147394623	0.0001235	c.124A>G	p.Lys42Glu	possibly damaging	Ashkenazi Jewish, European	(51)
(X) RF.FA.0715	Ashkenazi Jewish	Chr 1	26764719	*DHDDS (NM_205861*.*3)*	Homozygous	rs147394623	0.0001235	c.124A>G	p.Lys42Glu	possibly damaging	Ashkenazi Jewish, European	(51)
(Y) RF.KN.0216	Indian	Chr 4	16014922	*PROM1 (NM_006017*.*3)*	Heterozygous	rs137853006	NL	c.1117C>T	p.Arg373Cys	possibly damaging	NL	(52)

Note: NL: Not listed; N/A: Not applicable AF: Allele frequency. Co-segregation of all causative variants with disease has been confirmed (Table 2 and Fig 2).

This analysis detected *USH2A* variants as the underlying cause of disease in eight different pedigrees. Among these, only one was a novel variant while the remaining 15 were reported previously (Tables 1 and 2). The targeted mutation screening performed prior to WGS on a subset of cases did not include all currently known IRD genes nor cover all variants in a given gene; our current WGS screening resulted in the identification of variants in known genes in this set of pedigrees.

#### (iii) Structural Variants detected in known IRD genes

**Fig 3:**
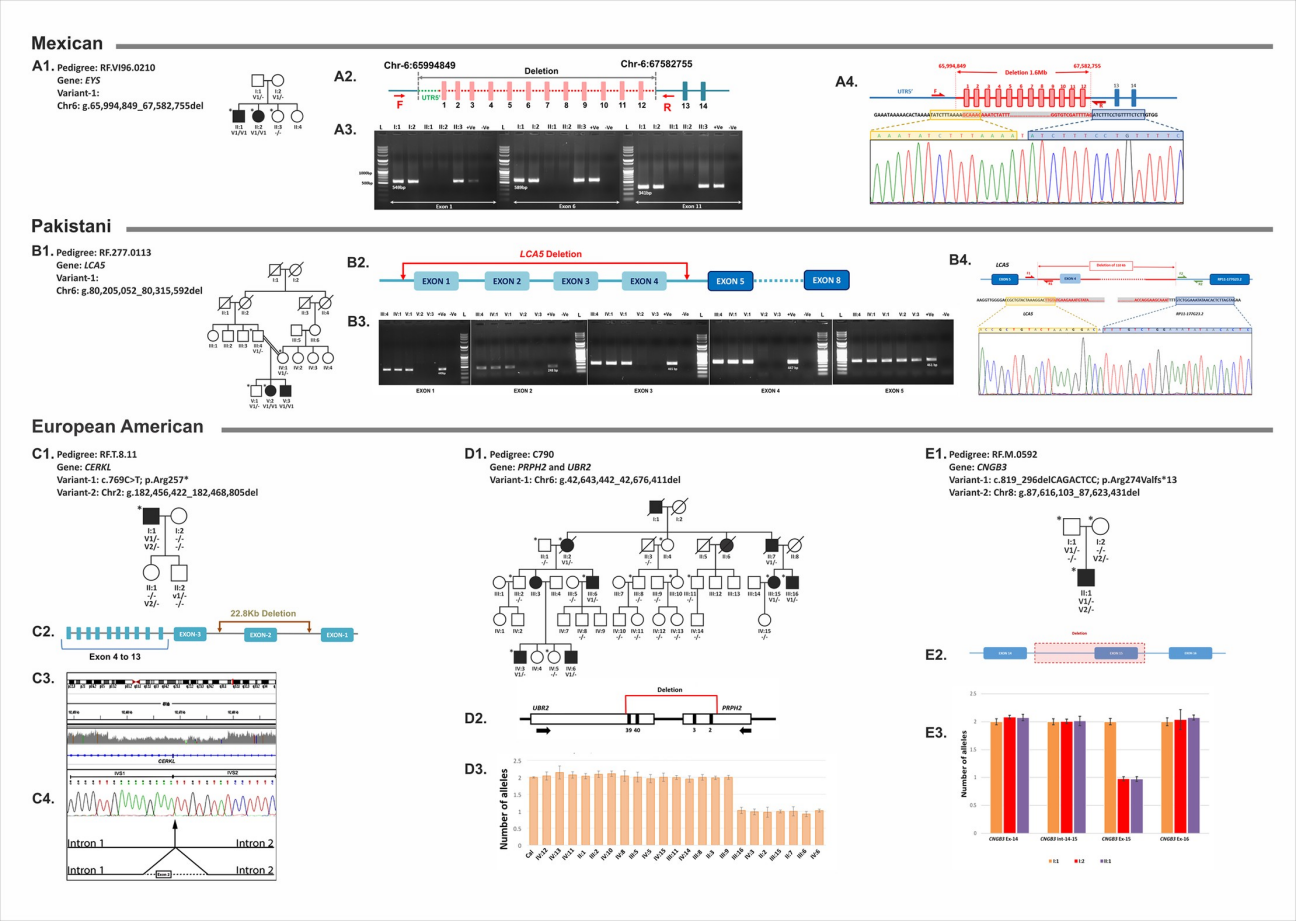
Pedigrees with copy number variations (CNVs). Five unique structural variants were identified in *EYS*, *LCA5*, *CERKL*, *PRPH2*, and *CNGB3* in five different pedigrees, one with dominant macular degeneration and four with recessive retinal degeneration. (A1) 1.6Mb homozygous deletion Chr6: g.65,994,849_67,582,755del is segregating with recessive retinal degeneration in a Mexican pedigree RF.V196.0210. (A2) The schematic diagram depicting a 1.6Mb homozygous deletion Chr6: g.65,994,849_67,582,755del encompassing the exons 1 to 12 and about 1.6Mb of 5’-untranslated region of the *EYS* gene. (A3) PCR amplification of EYS exons 1, 6, and 11 detected the presence of expected size product in unaffected individuals (I:1, I:2, II:3) whereas the presence of PCR product was not observed in two affected individuals (II:1 and II:2). (A4) Amplification with primers flanking the deleted region followed by sequencing revealed the junction point in individual II:1 due to the 1.6Mb deletion. Examination of the sequence flanking the junction point detected paralogous repeat sequences on both sides of the deleted region (blue and yellow boxes). (B1) In a consanguineous Pakistani pedigree RF.277.0113, a 110Kb homozygous deletion in *LCA5* (chr6: g.80,205,052_80,315,592del) segregated with the phenotype. (B2) The novel 110Kb homozygous deletion includes 1 to 4 exons of *LCA5*. (B3) PCR amplification showed the absence of exon 1 to 4 of *LCA5* in both affected individuals while exon 5 is present in all family members. (B4) Amplification with primers flanking the deletion resulted in the generation of the fragment with deletion. Sequencing this PCR product revealed the presence of paralogous repeat sequences flanking the junction point in affected individuals. Sequence marked with yellow and blue rectangles represent the paralogous sequence on both sides of the deleted region. (C1) A previously reported heterozygous stop mutation *CERKL* p.Arg257* and a novel heterozygous large 22.8 Kb deletion (Chr2: g.182,456,422_182,479,267del) on chromosome 2, which includes exon 2 of *CERKL* are observed in *trans* configuration in the proband of RF.T.8.11. (C2) The schematic diagram shows the 22.8Kb deletion which includes ~10.3Kb of intron 1 and exon 2 (243bp) and 12.3Kb of intron 2 of *CERKL* gene. (C3) The WGS reads mapped to the deleted region showed decrease in read depth. (C4) Electropherogram showing the sequence of junction fragment generated by amplification with primers flanking the deletion revealed the specific boundaries of the deletion that includes exon 2 of *CERKL*. (D1) The segregation analysis revealed a heterozygous 33Kb deletion on chromosome 6 (Chr6: g.42,643,442_42,676,411del) segregating with the disease. (D2) A cartoon depicting the deleted region which includes two different genes: exons 39 and 40 of *UBR2* and exons 2 and 3 of *PRPH2* present in opposite orientation (D3) Analysis of 7 affected and 15 unaffected members using qPCR confirmed the presence of the heterozygous deletion on chromosome 6 in affected members and not in unaffected relatives. (E1) A set of compound heterozygous deletions including a novel 7Kb deletion (Chr8: g.87,616,103_87,623,431del) and a previously known 7bp deletion p.Arg274Valfs*13 in *CNGB3* gene were observed in RF.M.0592 pedigree with a single affected individual. (E2) The novel 7Kb heterozygous deletion (Chr8: g.87,616,103_87,623,431del) (Pink rectangle) includes coding exon 15 of *CNGB3*. (E3) qPCR analysis confirmed the presence of the heterozygous deletion of *CNGB3* exon 15 in II:1, which was inherited from the mother (I:2). The asterisk indicates the availability of whole-genome sequencing data.

Five different pedigrees carried novel structural variants. These included one pedigree with dominant macular degeneration and the remaining four with recessive retinal degeneration. Of the recessive pedigrees, two had the novel structural variants in the homozygous state, one carried a previously reported nonsense mutation and one a previously reported frameshift mutation (Table 3 and Fig 3). Three of these pedigrees are European American while one each is Mexican and Pakistani.

**Table 3:** Pedigrees with copy number variations in IRD genes.

Pedigree	Ethnicity	Chr	Position	Gene (NM_ID)	Zygosity in affected individual	rsID	gnomAD AF	cDNA	Protein change	Population allele frequency (AF) (No of Homozygotes)	References
(A) RF.VI96.0210	Mexican	Chr 6	g.65,994,849_67, 582,755del	*EYS (NM_001142800*.*2)*	Homozygous	NL	NL	N/A	N/A	NL	This study
(B) RF.277.0113	Pakistani	Chr 6	g.80,205,052_80, 315,592del	*LCA5 (NM_001122769*.*3)*	Homozygous	NL	NL	N/A	N/A	NL	This study
(C) RF.T.8.11	European American	Chr 2	182423344	*CERKL (NM_201548*.*5)*	Comp Het	rs121909398	0.00039	c.769C>T	p.Arg257*	NL	(53)
			g.182,456,422_182,479,267del			NL	NL	N/A	N/A	NL	This study
(D) C790	European American	Chr 6	g.42,643,442_42, 676,411del	*UBR2 (NM_001363705*.*2) & PRPH2 (NM_000322*.*5)*	Heterozygous	NL	NL	N/A	N/A	NL	This study
(E) RF.M.0592	European American	Chr 8	87679178	*CNGB3 (NM_019098*.*5)*	Comp Het	rs775796581	0.00006012	c.819_826delCAGACTCC	p.Arg274Val fs*13	European (0.0001162), Finnish (0.00007964) (0)	(54)
			g.87,616,103_87, 623,431del								
						NL	NL	N/A	N/A	NL	This study

Note: Novel: Association with IRD has not been previously reported; NL: Not listed; N/A: Not applicable; AF: Allele frequency. Co-segregation of all causative variants with disease has been confirmed (Table 3 and Fig 3).

*(A) A 1*.*6Mb deletion in EYS segregating with IRD*. Analysis of the WGS of two affected (II:1 & II:2) and one unaffected sibling (II:3) from a Mexican pedigree RF.VI96.0210 (Fig 3A1) revealed a novel, 1.6 Mb homozygous deletion on chromosome 6 (Chr6: g.65,994,849_67,582,755del) in both affected members. This deletion was not observed in the unaffected sibling. The deleted region encompasses the exons 1 to 12 and 5’-untranslated region of the *EYS* gene implicated in recessive retinal degeneration (Fig 3A2). PCR amplification of exons 1 to 12 of *EYS* in this pedigree revealed the loss of exons in II:1 and II:2 (Fig 3A3). Amplification with primers flanking the deleted region followed by sequencing showed the overlapping of paralogous repeat sequences and deletion of in-between 1.6 Mb regions (Fig 3A4) in the affected members.

*(B) LCA5 gene deletion in a pedigree*. A 110Kb homozygous deletion in *LCA5* (Chr6: g.80,205,052_80,315,592del) was identified in a consanguineous Pakistani pedigree RF.277.0113. This homozygous deletion includes 1 to 4 exons of *LCA5*. PCR amplification and sequencing of the deleted region using primers located in the flanking sequence identified specific boundaries of the deletion and its segregation with *LCA* in RF.277.0113 (Fig 3B).

*(C) A 22*.*8Kb deletion in the CERKL gene*. In pedigree RF.T.8.11, a previously reported nonsense mutation p.Arg257* in the *CERKL* gene was identified in the heterozygous state by exome sequence analysis in the proband I:1 who was adopted (53). Whole-genome sequence analysis of this individual identified a large novel heterozygous 22.8 Kb deletion (Chr2: g.182,456,422_182,479,267del) on chromosome 2 (Fig 3C). Analysis of the samples of his two offspring established the compound heterozygous nature of the nonsense variant and the large deletion in the affected individual. The 22.8 Kb chromosome 2 deletion includes the entire coding sequence of exon 2 (243 bp) and about ~10.3 Kb of intron 1 (Chr2: g.182,456,422_182,468,805del) and 12.3 Kb of intron 2 (Chr2: g.182,468,565_182,479,267del) of the *CERKL* gene. The nonsense change is predicted to truncate the protein or result in nonsense-mediated decay (NMD) of the transcript [53]. The deletion of 22.8 Kb sequence encompassing exon 2 of *CERKL* may also result in the formation of a truncated protein due to coding region frameshift or the transcript may undergo NMD. Both sequence alterations detected in the *CERKL* gene in this individual are predicted to lead to the loss of functional protein; null mutations in *CERKL* have been established as the underlying cause of IRD [55, 56].

*(D) A large structural change involving two genes*. Clinical evaluation of eight affected individuals in a four-generation pedigree (C790) led to the diagnosis of autosomal dominant macular degeneration (MD) with no non-ocular abnormalities co-segregating with the MD phenotype (Fig 3D1). Analysis of WGS of five affected members and seven unaffected members revealed a heterozygous 33 Kb deletion on chromosome 6 (Chr6: g.42,643,442_42,676,411del) in affected members and not in unaffected relatives. This deletion included two adjacent genes present in opposite orientation: exons 39 and 40 of *UBR2* and exons 2 and 3 of *PRPH2* (Fig 3D2). Segregation analysis of 7 affected and 15 unaffected members using qPCR confirmed the segregation of the chromosome 6 deletion with the phenotype (Fig 3D3). The *PRPH2* gene alterations including loss of function mutations have been implicated in dominant MD [57] and other retinal dystrophies, while the *UBR2* gene is not associated with IRD or any other pathological condition.

In addition to the large deletion, two affected (IV:3 & IV:6) and one unaffected (IV:5) offspring of an affected female (III:3) were observed to carry a rare heterozygous potentially pathogenic variant c.659T>G, p.Phe220Cys (Allele frequency in gnomAD = 0.00002) in the rhodopsin gene. While samples of the parents of these individuals were not available for genetic analysis, the novel rhodopsin variant c.659T>G, p.Phe220Cys was not detected in either maternal grandparents (II:1 & II:2) suggesting the possible paternal (III:4) inheritance of this variant in the three siblings (IV:3, IV:5 & IV:6). Further, this variant was not detected in the rest of the pedigree excluding the possible involvement of c.659T>G, p.Phe220Cys as the variant responsible for IRD pathology in the rest of the extended pedigree. The two affected individuals IV:3 and IV:6 have both the large deletion encompassing *UBR2* and *PRPH2* and the c.659T>G in the rhodopsin gene. The impact of having both sequence alterations in these individuals is unknown.

*(E) Compound heterozygous deletions in CNGB3*. Whole-genome sequence analysis of pedigree RF.M.0592 with a single affected individual identified a novel 7Kb heterozygous deletion (Chr8: g.87,616,103_87,623,431del) and an additional previously known 7bp heterozygous indel (c.819_826delCAGACTCC) in *CNGB3* gene that results in p.Arg274Valfs*13 [54]. The frameshift mutation was inherited from the father while the large deletion was inherited from the mother (Fig 3E).

Moreover, our analysis identified deletions and sequence alterations in non-coding regions with either unknown impact or yet to be annotated. As state above, experimental and analysis approaches will need to be developed to validate this class of potential disease-causing mutations.

#### (iv) Atypical genotypes observed in IRD pedigrees

**Fig 4:**
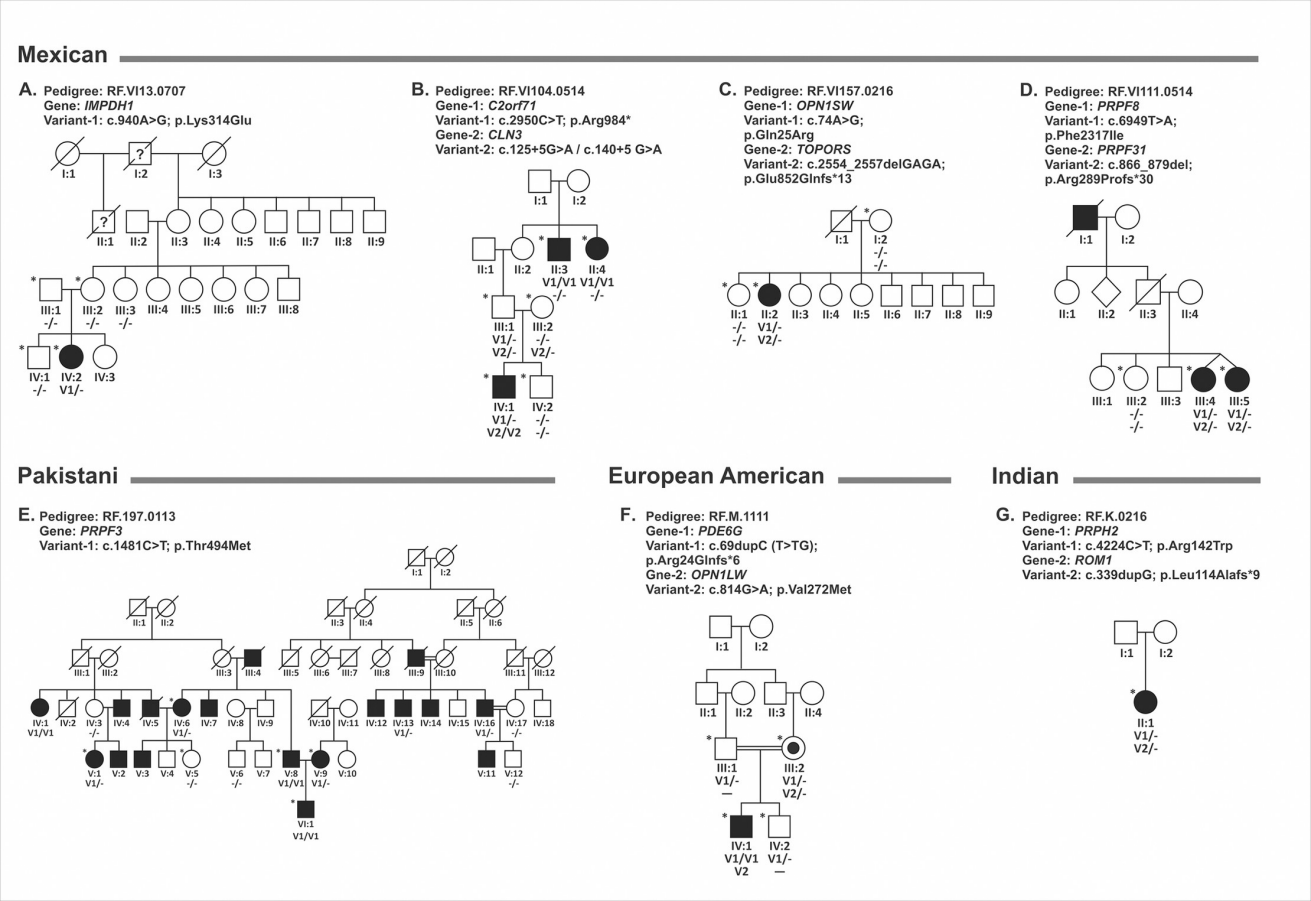
Segregation analysis of pedigrees with atypical genotypes. A-D: Mexican pedigrees with atypical mutations. **A.** RF.VI13.0707 pedigree with a de-novo mutation in *IMPDH1*; **B.** RF.VI104.0514 pedigree with a homozygous nonsense mutation in *C2orf71* was detected in generation II while a previously reported homozygous splice site mutation in the *CLN3* gene was observed in proband in the IV generation demonstrating the involvement of two different genes in IRD pathology in different generations. **C.** RF.VI157.0216 pedigree with mutations in genes *OPN1SW* and *TOPORS* associated with dominant color blindness and retinitis pigmentosa; **D.** RF.VI111.0514 pedigree with novel heterozygous causative mutation in *PRPF8* and a known mutation in *PRPF31*, each sufficient to cause dominant IRD, were observed in monozygotic affected twins; **E.** RF.197.0113 consanguineous pedigree from Pakistan with a previously known dominant acting mutation in *PRPF3* segregated in a pseudo-recessive pattern; **F**. RF.M.1111 an European American pedigrees with causative variants in more than one known IRD genes were observed to segregate with disease. A homozygous mutation in *PDE6G* and a hemizygous mutation in *OPN1LW* were observed in Pedigree RF.M.1111. **G.** RF.K.0216 Indian pedigree with a heterozygous *PRPH2* mutation that is sufficient to cause retinal dystrophy and an additional mutation in *ROM1* that can lead to digenic RP along with the *PRPH2* variant. The asterisk indicates the availability of whole-genome sequencing data.

Analysis of the WGS identified atypical causal variants in 7 pedigrees including four pedigrees from Mexico, one from Pakistan, one European American and one of India origin (Table 4 and Fig 4).

**Table 4:** Pedigrees with atypical genotypes in IRD genes.

Pedigree	Ethnicity	Chr	Position	Gene (NM_ID)	Zygosity in affected individual	rsID	gnomAD AF	cDNA	Protein change	Prediction by PolyPhen2	Known/Novel	Population listing in gnomAD (AF) {ClinVar}	References
(A) RF.VI13.0707	Mexican	Chr 7	128038602	IMPDH1 (NM_000883.4)	Heterozygous De-novo	NL	NL	c.940A>G	p.Lys314Glu	probably damaging	novel	NL	This study
(B) RF.VI104.0514	Mexican	Chr 2	29294178	C2orf71 (NM_001029883.3)	Homozygous	rs774215025	0.00001655	c.2950C>T	p.Arg984*	N/A	known	Latino (0.0002317), European (0.000008872); (0)	(58, 59)
		Chr 16	28502798	CLN3 (NM_001042432.2)	Homozygous	rs386833704	0.00001596	c.125+5G>A / c.140+5 G>A	N/A	N/A	known	Latino (0.0001158); (0)	(60)
(C) RF.VI157.0216	Mexican	Chr 7	128415771	OPN1SW (NM_001385125.1)	Heterozygous	NL	NL	c.74A>G	p.Gln25Arg	probably damaging	novel	NL	This study
		Chr 9	32541965	TOPORS (NM_005802.5)	Heterozygous	rs527236116	0.00003191	c.2554_2557delGAGA	p.Glu852Glnfs*13	N/A	known	European (0.00006494); (0)	(61)
(D) RF.VI111.0514	Mexican	Chr 17	1554155	PRPF8 (NM_006445.4)	Heterozygous	NL	NL	c.6949T>A	p.Phe2317Ile	probably damaging	novel	NL	This study
		Chr 19	54629912	PRPF31 (NM_015629.4)	Heterozygous	NL	NL	c.866_879delGGAAAGCGGCCCGG	p.Arg289Profs*30	N/A	known	NL	(32)
(E) RF.197.0113	Pakistani	Chr 1	150316692	PRPF3 (NM_004698.4)	Heterozygous	rs121434241	NL	c.1481C>T	p.Thr494Met	probably damaging	known	NL	(62, 63)
(F) RF.M.1111	European American	Chr 17	81653236	PDE6G (NM_002602.4)	Homozygous	NL	NL	c.69dupC (T>TG)	p.Arg24Glnfs*6	N/A	known	NL	(20)
		Chr X	153421838	OPN1LW (NM_020061.6)	Hemizygous	NL	NL	c.814G>A	p.Val272Met	probably damaging	novel	South Asian (0.00005288), Latino (0.00003657); (0) {ClinVar: NL}	This study
(G) RF.K.0216	Indian	Chr 6	42689649	PRPH2 (NM_000322.5)	Heterozygous	rs61755783	0.00002122	c.424C>T	p.Arg142Trp	probably damaging	known	Latino (0.00008465), European (0.00002323); (0)	(61, 64)
		Chr 11	62381083	ROM1 (NM_000327.4)	Heterozygous	rs137955062	NL	c.339dupG	p.Leu114Alafs*9	N/A	known	NL	(65)

Note: Novel: Association with IRD has not been previously reported; NL: Not listed; N/A: Not applicable, AF: Allele frequency. ClinVar classification provided for all novel variants. Co-segregation of all causative variants with disease has been confirmed (Table 4 and Fig 4).

**Fig 5:**
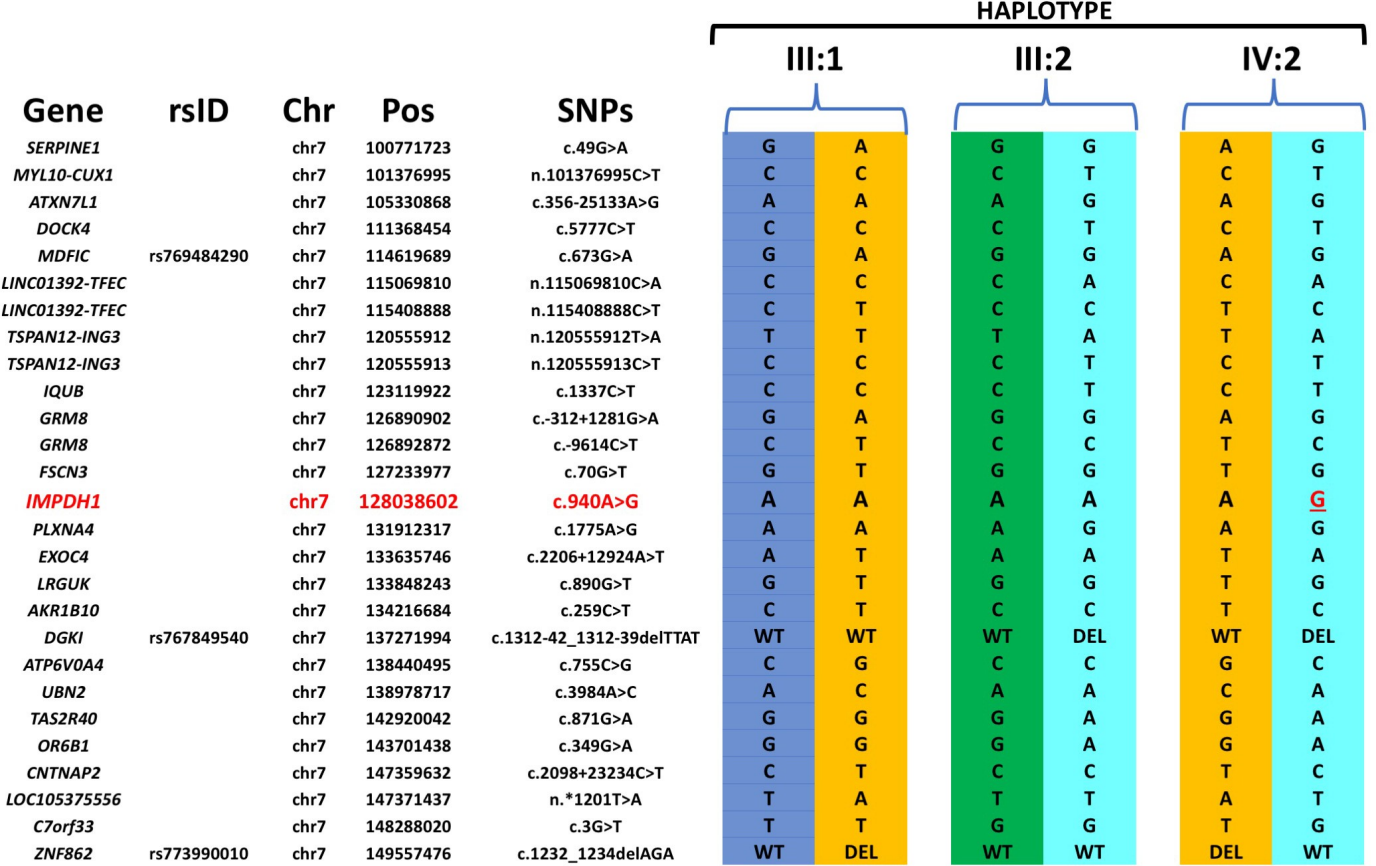
Haplotype of parents and the proband constructed with variants flanking the de-novo variant detected in the *IMPDH1* gene in RF.VI13.0707 pedigree (Fig 4A).

- Analysis of a Mexican pedigree RF.VI13.0707 with one affected and four unaffected members available for the study detected a de-novo novel heterozygous potentially pathogenic variant c.940A>G, p.Lys314Glu in the *IMPDH1* gene associated with autosomal dominant RP (Fig 4A). The clinical changes observed in the affected individual are consistent with *IMPDH1* associated retinitis pigmentosa with marked macular atrophy (S1A Fig). Analysis of the WGS data of the proband, parents and the unaffected sibling using identical by descent (IBD) segment analysis established the genetic relatedness and verified the provided family structure. Examination of the haplotypes of parents and the proband that were constructed using variants in the region encompassing the *IMPDH1* gene confirmed the shared haplotype between parent and offspring (Fig 5). However, the absence of the novel c.940A>G, p.Lys314Glu in the *IMPDH1* variant in either parent was noted establishing the c.940A>G change as a de-novo variant (Fig 5) and suggesting it as the possible underlying cause of IRD in this patient. The maternal great grandfather (I:2) and grand uncle (II:1) of the patient were reported with vision loss but it is not known if they had a clinical phenotype of RP nor was genotyping of these individuals possible.
- The WGS variants data set of three affected members (II:3, II:4, and IV:1) and three unaffected members (III:1 and III:2 and IV:2) of a four-generation Mexican pedigree RF.VI104.0514 was analyzed. WGS analysis identified two potentially pathogenic variants in two separate genes in affected individuals from different generations. Two affected siblings II:3 and II:4 were observed to carry a homozygous nonsense variant c.2950C>T; p.Arg984* in *C2orf71*, which has previously been reported as a mutation causing recessive RP [58, 59]. However, this mutation was detected only in the heterozygous state in the affected male IV:1 as well as in his unaffected father. Further analysis of sequence variants revealed an additional previously reported homozygous splice site mutation c.125+5G>A (c.140+5G>A) in the *CLN3* gene in IV:1, but not in other affected members II:3 and II:4 [60]. This variant was also observed in the heterozygous state in his unaffected parents III:1 and III:2. Both *C2orf71* and *CLN3* gene variants segregated with disease in separate branches of the RF.VI104.0514 pedigree (Fig 4B). Patient IV:1 was examined at the age of 10 years with a report of lipofuscinosis, which is consistent with the *CLN3* mutation detected [66]. The age of onset in all three affected members IV:1, II:3, and II:4 is reported to be during early childhood (4-5yrs). But the fundus images of II:3 and II:4 at a younger age are not available. Individuals II:3 and II:4 who are currently in their 80s are likely affected with recessive RP due to the *C2orf71* mutation (S1F to S1H Fig) while IV:1 has subtle macular changes due to the *CLN3* variant. Best-corrected visual acuities were only 20/200 and 20/100 at the age of 10 years and the patient was noted to have major mood disturbance as well as a very serious change in personality leading to a referral to a neurologist.
- In the RF.VI157.0216 Mexican pedigree with a single affected member, the WGS analysis revealed the presence of two pathogenic novel heterozygous pathogenic variants in two different genes *OPN1SW* (c.74A>G, p.Gln25Arg; Chr 7) and *TOPORS* (c.2554_2557delGAGA, p.Glu852Glnfs*13; Chr 9) associated with dominant color blindness (Tritenopia) and dominant retinitis pigmentosa respectively [67–69]. The age of onset of IRD in the proband (II:2) was between 6–7 years. This patient is diagnosed with Marfan syndrome and multiple sclerosis and reported color deficiency since the age of 17 years. While the color deficiency is consistent with the involvement of *OPN1SW* mutations, the retinal degeneration phenotype in this individual is consistent with the phenotype associated with *TOPORS* (Fig 4C).
- RF.VI111.0514 Mexican pedigree includes affected fraternal twins and an affected grandparent. WGS analysis of the affected twin sisters (III:4 & III:5) and their unaffected sibling (III:2) identified novel heterozygous damaging variant in *PRPF8* (c.6949T>A; p.Phe2317Ile, Chr 17) and a known heterozygous *PRPF31* (c.866_879delGGAAAGCGGCCCGG; p.Arg289Profs*30, Chr 19) mutation in affected twins and not in their unaffected sibling [32]. Sanger sequencing further confirmed these findings (Fig 4D). Either of these mutations are sufficient to cause the RP phenotype observed in affected members (S1I Fig). The presence of macular cysts and the retinal phenotype observed in affected twins is more consistent with the phenotype associated with *PRPF31* than with *PRPF8*. The paternal grandfather (I:1) is reported to be affected with IRD, while the clinical status of the father (II:3) is unknown.
- A large Pakistani pedigree, RF.197.0113 with 5 consanguineous marriages, and eight affected members available for the study was analyzed. Considering recessive inheritance, the WGS data of individuals IV:6, V:1, V:5, V:8, V:9 and VI:1 for homozygous potentially damaging variants shared between affected members and not present in unaffected member did not reveal candidate causative variants segregating with the disease. Subsequently, considering the dominant inheritance, the WGS variants of six individuals were filtered for potentially damaging heterozygous variants shared by all affected members and absent in the homozygous or heterozygous state in unaffected members. The latter analysis also did not identify candidate variants segregating with the disease. Further filtering for all potentially damaging variants present in either a heterozygous or homozygous state detected a previously reported adRP-associated variant, c.1481C>T, p.Thr494Met in the *PRPF3* gene in five affected members in the heterozygous state, in three in the homozygous state and none of the unaffected relatives. Analysis of all members of this pedigree for this variant revealed the segregation of the c.1481C>T *PRPF3* with the disease in the pedigree RF.197.0113 in a pseudo-recessive pattern due to the consanguinity (Fig 4E).
- A consanguineous European American pedigree RF.M.1111 (Fig 4F) with an affected male with a diagnosis of typical retinitis pigmentosa and an unaffected brother were analyzed by WGS of the two siblings (IV:1 and IV:2) and parents (III:1 and III:2). Analysis of variants in these individuals detected a homozygous novel c.69dupC, p.Arg24Glnfs*6 variant in *PDE6G* segregating with retinitis pigmentosa phenotype. In addition, IV:1 carried the hemizygous variant in *OPN1LW* (c.814G>A, p.Val272Met) gene on the X chromosome inherited from the mother (III:2). The clinical symptoms reported in the affected individual are more consistent with severe RP phenotype associated with *PDE6G* mutations; color vision was not tested in this individual.
- In the RF.K.0216 pedigree (Fig 4G) from India, two previously known dominant mutations, one in *PRPH2* (c.424C>T, p.Arg142Trp) [61, 64, 70] and another in *ROM1* (c.339dupG, p.Leu114Alafs*9) [65, 71] were observed in a patient with a diagnosis of central areolar choroidal dystrophy (CACD) with onset in the 5^th^ decade and mild central vision loss consistent with the phenotype associated with the p.Arg142Trp mutation in *PRPH2* [72]. The impact on this patient of the additional *ROM1* mutation p.Leu114Alafs*9 mutation is unknown.

In summary, a novel de-novo causative variant c.940A>G, p.Lys314Glu in the *IMPDH1* gene associated with autosomal dominant RP was observed in one pedigree (RF.VI13.0707); and a previously known dominant mutation in *PRPF3* (c.1481C>T) was detected in RF.197.0113 in a pseudo-recessive pattern due to multiple consanguineous marriages. In addition, potentially pathogenic variants in two independent genes both segregating with the disease and each sufficient to cause pathology were detected in four out of the 7 pedigrees with atypical genotypes. Besides these 7 pedigrees, we previously reported the identification of mutations in two independent genes as the underlying cause of IRD in separate branches of a pedigree by WGS in a European American pedigree [11].

#### (v) Variants described in recently demonstrated novel IRD genes by our group

WGS sequence analysis detected potentially pathogenic candidate causative variants in genes previously not associated with IRD segregating with disease in two pedigrees in this cohort and we have reported these findings earlier [10, 14]. Our genetic and functional evaluation of these genes established the involvement of *AGBL5* and *IFT88* in causing IRD in the two unrelated pedigrees [10, 14].

#### (vi) Classification of Clinical Phenotypes based on WGS analysis findings

The initial clinical diagnosis of pedigrees spanned a broad spectrum including RP in 18, cone dystrophy in 1, macular dystrophy in 1, Leber congenital amaurosis (LCA) in 2, Usher syndrome in 2, with the majority (37) having unclassified retinal degeneration (S1 Table). Re-evaluation of clinical data in the context of our genetic analysis findings lead to the reclassification of clinical phenotypes in our cohort: RP in 45 pedigrees, cone dystrophy in 7, LCA in 3, congenital stationary night blindness in 1, and macular dystrophy, nephronophthisis, Ceroid lipofuscinosis, choroideremia and Usher syndrome in one family each (S1 Table).

#### (vii) IRD Causative mutations detected in three populations studied

**Fig 6:**
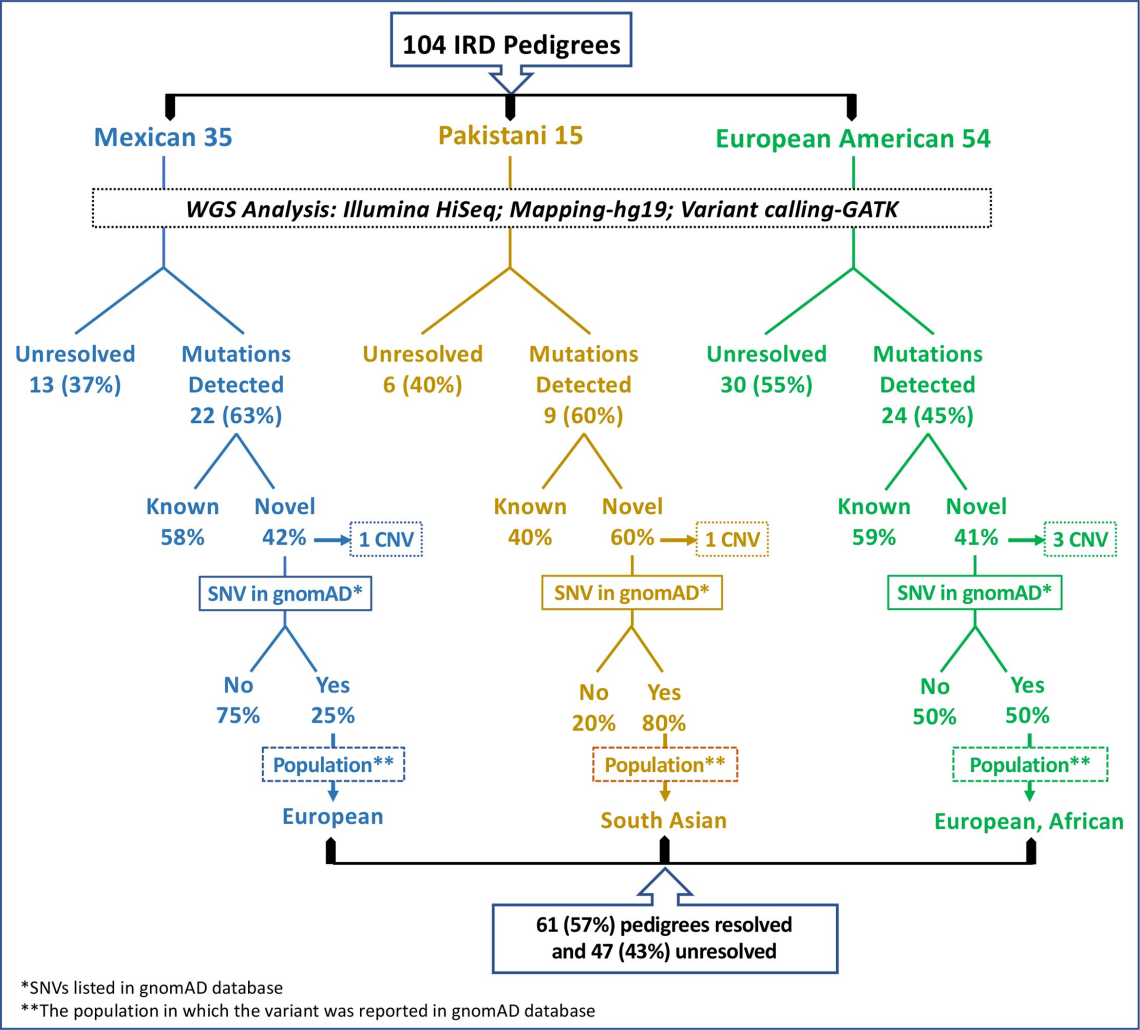
Summary of findings and population distribution of novel mutations detected in Mexican, Pakistani and European American pedigrees.

*(A) Analysis of pedigrees from Mexico*. In the current study, WGS analysis of 35 pedigrees with recessive retinal dystrophy excluding STGD1 detected 18 previously reported and 13 novel (42%) causative mutations in known IRD genes in 22 pedigrees (63%) leaving the remaining 13 pedigrees unresolved (Fig 6). Nine (75%) of the 12 novel mutations involving SNVs observed in cases from Mexico were not listed in the gnomAD database while the remaining are reported only in the Latino population as very rare variants (Tables 1, 2, 3, 4 and 5). Mutations in *USH2A* are the most frequent cause of recessive retinal degeneration in this population with four pedigrees from the current study (RF.VI148.1215, RF.VI145.1215, RF.VI129.0714, and RF.VI127.0514) and two additional pedigrees from our previous studies with causative mutations in *USH2A* [4]. Female carriers of RPGR mutations in two pedigrees (RF.VI123.0514 and RF.VI153.0216) developed retinal degeneration phenotype as reported earlier [73].

*(B) Analysis of pedigrees from Pakistan*. In this study, we have analyzed 15 consanguineous Pakistani pedigrees with multiple affected members and identified causative mutations in 9 IRD genes in 9 pedigrees (60%) while the causative mutations were not detected in 6 pedigrees (40%) (Fig 6). Among the causative mutations detected in IRD associated genes, 6 are novel (60%) and 4 are previously reported (Fig 6 and Table 5). Four of the 5 novel mutations involving SNVs detected were reported in gnomAD database as extremely rare variants in South Asians, one in Europeans and the remaining one (~20%) was not listed (Tables 1, 2, 3, 4 and 5).

**Table 5:** Summary of findings and population distribution of mutations detected in three major cohorts analyzed.

**Mexican population:**	
Number of pedigrees	35
Origin	Native Indian + European
Mutation detection rate	63% (22/35)
Novel IRD mutations	42%
Novel IRD SNVs not in gnomAD	75% (never reported before)
Novel IRD mutations listed in gnomAD	Specific to Europeans
**Pakistani population:**	
Number of pedigrees	15
Origin	Asian-Endogamous
Mutation detection rate	60% (9/15)
Novel IRD mutations	60%
Novel IRD SNVs not in gnomAD	20% (never reported before)
Novel IRD mutations listed in gnomAD	Specific to South Asians
**European American population:**	
Number of pedigrees	54
Origin	European
Mutation detection rate	45% (24/54)
Novel IRD mutations	41%
Novel IRD SNVs not in gnomAD	50% (never reported before)
Novel IRD mutations listed in gnomAD	European and African

Note: Population distribution of novel variants listed in gnomAD database is included in Tables 1, 2, 3 and 4.

*(C) Analysis of pedigrees from the United States*. Fifty-four pedigrees, which comprise 50% of the total analyzed in this study are of European ancestry. Causative mutations were detected in 24 (45%) pedigrees that included 25 known mutations (~59%), 14 novel single nucleotide changes (SNVs) and 3 novel structural changes (Tables 1, 2, 3, 4 and 5). Seven (~50%) of the 14 novel SNVs in known IRD genes, were not listed in the gnomAD database (Table 5). Two variants, c.722-1G>T in *GUCY2D* and c.1217G>A variant in *CNGB1*, detected in our European American cohort were reported in the African population at low frequency (gnomAD database), while the remaining appear to be unique to the European population (Fig 6 and Table 5).

*(D) Pedigrees of Indian ancestry*. Two pedigrees recruited in the United States are of Indian origin in which three previously reported causative mutations were detected (Tables 2 and 4).

*(E) Pedigrees of Ashkenazi Jewish ancestry*. In this study a previously known homozygous p.Lys42Glu mutation in *DHDDS* was detected in two Ashkenazi Jewish pedigrees recruited in the United States (Table 2).

Overall, the underlying cause of IRD was identified in about 57% of pedigrees. However, the rate of causative mutation identification in Mexican (63%), Pakistani (60%) and European American (45%) pedigrees varied. The number of novel IRD causative mutations detected in each of these cohorts also varied from about 42% and 41% in both Mexican and European American pedigrees to 60% in Pakistani pedigrees. Further, among the novel IRD causative SNVs, 20% of those detected in Pakistani pedigree were not listed in gnomAD database while 50% and 75% of novel SNVs in European American and Mexican pedigrees were not in the gnomAD database (Table 5).

## Discussion

Analysis of the whole-genome sequence of this cohort comprised of 404 individuals from 108 pedigrees with inherited retinal degeneration identified 93 causal variants in 232 individuals in 61 (57%) pedigrees. Among the causative variants detected, 39 (42%) are novel and 54 (58%) are previously reported variants in 44 well established IRD associated genes and two IRD genes we recently reported [10, 14]. Although a majority of pedigrees underwent prior screening for mutations in known genes without success, WGS analysis identified causative variants in IRD genes. This is primarily due to the limitations in the mutation screening panels used over the past two decades that did not include many currently known IRD associated genes. Further, the early version of exome capture probes that did not cover complete coding sequences. Variants in novel genes or variants in non-coding regions of known IRD genes with unknown impact or yet to be annotated may contribute to the phenotype in the 47 pedigrees that remained unresolved in this study.

The outcomes of the analysis of 108 IRD pedigrees provided insight into the genetic architecture of IRD. Overall novel mutations were identified in genes known to be associated with IRD in 36 pedigrees while previously reported mutations were detected in 25 pedigrees. The majority of the mutations (60%) were missense mutations including stop gain variants, 23% frameshift, while only 5% were structural variants and 12% were potential splice altering variants. All the causative CNVs detected in this study were novel. Analysis of the sequence flanking these deletions revealed microhomologies suggesting potential non-homologous end-joining leading to these deletions (Fig 3). Atypical genotypes were detected in a set of pedigrees (12%). These included causative mutations in more than one gene that segregated with IRD. While causative mutation(s) in one gene is potentially sufficient to explain pathology, the impact of having an additional causative mutation in a second IRD gene is unknown due to the significant overlap in the phenotype of IRDs. Further, intrafamilial genetic heterogeneity was observed in one pedigree. Such cases reveal the need for a comprehensive analysis of all known IRD genes for molecular diagnosis, counseling, and particularly for treatment decisions. In several cases, heterozygous pathogenic variants were also detected in IRD genes in several cases in addition to the primary causative mutations. A deeper phenotype-genotype analysis on a larger cohort, in the context of additional pathogenic variants, may provide further insight into variation in the IRD phenotype and molecular pathology of IRD. The occurrence of de-novo mutations is rare in retinal disease genes [74–77] and a heterozygous de-novo mutation in *IMPDH1* was detected in one affected individual in our cohort. This is the first report of a de-novo variant in the *IMPDH1* gene.

It is interesting to note that only a small proportion of novel causative genes were identified despite a significant proportion of our pedigrees originating from understudied populations. Further, the two novel genes observed to carry causative mutations in our cohort were detected in small pedigrees of European Americans [10, 14]. The low number of novel IRD causative genes detected is consistent with the low number of novel IRD genes reported in the literature in the past few years [2]. An exponential increase in novel IRD gene discovery occurred in two majors spurts between 2000–2005 and 2010–2015 [2]. The spurts coincided with the development of advanced genome analysis tools and consequent enhancement in our knowledge of the architecture of the genome. Continuing with this trend, recent studies revealed the contribution of atypical genomic changes in IRD genes to pathology [78–80]. Our findings are consistent with the observation that the discovery of novel IRD genes is approaching a plateau phase and atypical genomic alterations in known IRD genes may contribute to about 10%-15% of cases [12, 79]. The number of unrelated pedigrees with mutations in recently identified novel IRD genes, both in our studies and in the literature is small suggesting these mutations could be more recent or private and are not major contributors to IRD. The underlying cause of pathology in 47 (43%) pedigrees that remained unresolved in our cohort after WGS may also involve atypical genotypes including alterations in non-coding sequences or in regions of the genome that are not well understood [80–82]. Therefore, gaining a deeper understanding of the genome, particularly the impact of non-coding variants, may improve our understanding of the molecular architecture of IRD and help resolve the remaining cases. Further advances in genome analysis methodologies may also facilitate the detection of the molecular cause of IRD in these unresolved pedigrees.

The families analyzed in this study included families that are primarily from understudied populations from Pakistan and Mexico and a third, well-studied European American population. About a third of the pedigrees included in this study are from Mexico with a unique population in which the genetics of IRD are not well understood. Comprehensive genetic analysis of IRD in this population has been reported primarily in two publications including one of our own [4, 32, 83–85]. Our previous analysis of 6 Mexican pedigrees from this region using whole-exome sequencing detected 3 novel and 6 known causative variants in IRD associated genes [4]. Zenteno et al described targeted genetic analysis of a cohort of probands with IRD and detection of mutations in 66% of cases with 48% of these mutations being novel [32]. The current analysis of 35 pedigrees using the WGS detected causative mutations in 63% of pedigrees from Mexico and 42% of these are novel. These findings are similar to the observations reported in the prior two publications and reflect the understudied nature of this population [4, 32]. Further, 75% of these novel potentially pathogenic SNVs detected in our study are not listed in the gnomAD database. Since the Mexican population is an admixture of indigenous peoples and individuals of European ancestry [86, 87]; the detection of a large proportion of novel variants not listed gnomAD may be due to their possible origin from the indigenous population in Mexico that are not well represented in gnomAD data set.

The second population included in our analysis is from the Punjab province of Pakistan. Until recently, the genetics of IRD in this population was not well studied. The structure of the Pakistani population is unique with endogamous sub-populations of multi-ethnic origin and high consanguinity in each of these populations [88–90]. Our earlier studies on 208 multigenerational pedigrees from the same region with a diagnosis of recessive IRD [7, 91–104] found homozygous causative mutations in 149 pedigrees (~71%). So far, mutations in novel genes were observed in only five (2.5%) unrelated Pakistani pedigrees in our cohort *ASRGL1* [99], *IFT43* [104], *ZNF513* [105], *SLC24A1* [106], and *CLCC1* [93]) while the remaining resolved pedigrees (97.5%) had mutations in known IRD genes. Among the mutations detected in known genes, p.Pro363Thr in *RPE65* is the most common causative mutation found in this population [7, 91, 107]; this variant was observed only in the South Asian population (gnomAD database). An independent study on a cohort of Pakistani families also reported 70% novel and 30% previously identified variants in IRD associated genes [108–122]. Consistent with these findings, causative mutations were detected in 60% of pedigrees in the current study cohort with 60% of the mutations being novel. However, the majority of these novel IRD associated SNVs were listed in the South Asian population in the gnomAD database (7 out of 8) unlike the novel SNVs in the Mexican population.

Interestingly, the mutation detection rate was lower (45%) in European American pedigrees compared to the rate in Mexican and Pakistani pedigrees (63% and 60%, respectively). Despite the well-studied nature of this population, 41% of the mutations detected in this study cohort are novel. Furthermore, 50% of these novel causative SNVs are not listed in gnomAD database.

**Fig 7:**
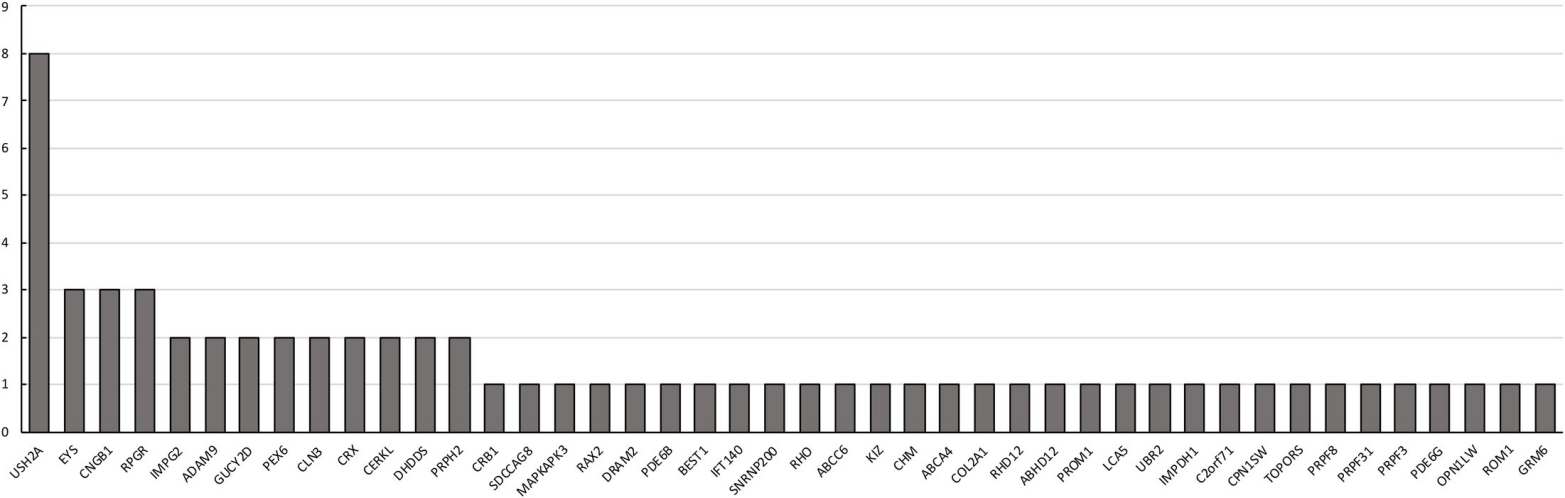
Distribution of IRD genes detected in 61 pedigrees.

Overall, *USH2A* is most frequently associated with IRD followed by *EYS*, *CERKL*, *CRX*, *IMPG1* and *RPGR* in the current study cohorts (Fig 7). Studies describing the genetic analysis of IRD in geographically distinct populations using a range of methods have been reported [12, 32, 123–132]. These studies found *USH2A* as the gene frequently associated with recessive RP worldwide including the European, Mexican and Pakistani populations [12]. In addition, the involvement of selected genes including *EYS*, *RPE65*, *CEP290* in IRD is reported at higher frequency in certain populations [133]. Further, the involvement of *ZNF513* and *INPP5E* in IRD is reported only in Pakistani and European populations respectively [134]. Population specific founder mutations have also been reported [135]. Our previous studies on Pakistani population identified p.Pro363Thr variant in *RPE65* that is specific to the South Asian population as the common causative mutation [7, 92]. The distribution of potentially causative variants detected in the study cohort is consistent with findings on other populations. Although the Pakistani population and some of the sub-populations in Mexico are endogamous in nature, the occurrence of causative variants at higher frequency is not observed in these populations compared to other populations.

The majority of novel mutations identified in our cohort are either not listed in the gnomAD database or observed at very low frequency in Latino (for the Mexican), South Asian (for the Pakistani), or European (for the European American) populations (Tables 1, 3, 4 and 5). It is unknown if the novel variants detected in cases from the Mexican population are more recent variants in the Latino population or have originated from the indigenous population which might not be well represented in gnomAD data. Similarly, all the novel causative variants found in the Pakistani cohort are either absent or occur at very low frequency in the South Asian population suggesting those to be unique to this population. Further, these were observed only in one or a few Pakistani pedigrees despite the endogamous nature of this population. Surprisingly, a similar trend was observed with the novel mutations detected in the well-studied American population. Eight out of 21 novel mutations detected in European American pedigrees including *AGBL5* and *IFT88* variants were not listed in gnomAD while the remaining are specific to European population. These findings suggest that the novel mutations detected in our cohort are possibly specific to their corresponding populations or private mutations, particularly the ones observed in European Americans. A majority of pedigrees analyzed in the current study were prescreened for mutations utilizing targeted mutation screening methodologies designed based on data predominantly from European Americans [136–138]. This bias has possibly contributed to the detection of high proportion of novel causative variants, particularly in the set of European American pedigrees. Overall, the findings on geographically diverse and understudied Mexican and Pakistani populations and the well-studied Caucasian population including our own data revealed that the pattern of distribution of IRD causative mutations in this cohort was similar to the findings reported in other worldwide populations. As the number of pedigrees studied from each ethnic group is small, analysis of additional IRD cases from the understudied Pakistani and Mexican populations may provide better insight into the genetic architecture of these populations. Further, appropriate classification of the clinical relevance of novel potentially causative variants using population specific information and the impact of the corresponding gene will facilitate improved genetic diagnosis to patients from worldwide populations [139].

This study using WGS and in-depth integrated analysis of the nature and type of mutations in different populations, provided insight into the population-specific genetic architecture of IRD and enabled it’s comparison to other worldwide populations. Such information will be helpful in the design of efficient population-specific tools for molecular diagnosis, genetic counseling, and decision on the selection of therapies. Further analysis of the 47 pedigrees that remained unresolved in this study may lead to the identification of causative variants in novel genes or non-coding variants that can contribute to the phenotype by modifying enhancer-promoter interactions or other yet to be identified functions of non-coding sequences.

## Methodology

### Ethics statement

The study protocol adhered to the tenets of the declaration of Helsinki and was approved by the Institutional Review Boards of the University of California San Diego, USA; University of California San Francisco, USA; University of Michigan, Kellogg Eye Center, USA; Johns Hopkins University School of Medicine, USA; University of Arizona, USA; Retina and Genomics Institute, Yucatán, México; Genetics and Ophthalmology, Genelabor, Goiânia, Brazil and University of Punjab, Lahore, Pakistan. Preliminary information on the clinical history of the patients and their family members were collected for the study along with the family history. Blood samples were collected from all available family members after obtaining their written consent to participate in our study.

#### Pedigree selection

Pedigrees with at least one individual with a diagnosis of non-syndromic inherited retinal degeneration (IRD) were recruited. Patients with a primary diagnosis of Stargardt (STGD1) were excluded from this study. Self-reported ethnicity information was recorded.

#### Patient samples

A total of four hundred and nine individuals from 108 unrelated families were analyzed by performing whole-genome sequencing. Among these, 203 individuals were affected and 206 were unaffected with 206 females and 203 males. 15 families were recruited from Pakistan, 35 from Mexico, 2 from India, 2 were Ashkenazi Jewish, and the remaining 54 families were of European ancestry from the USA.

The 108 pedigrees in this study had previously been examined for mutations in known IRD genes using a wide range of methodologies available. A set of 31 pedigrees with 1 to 5 affected members were previously analyzed by sequencing whole-exomes of selected members using Nimblegen V1-V3 (Roche Nimblegen, Inc., Wisconsin) or Agilent V1-V5 + UTRs probes (Agilent Technologies, Santa Clara, CA) to identify disease-causing gene mutations but remained unresolved. Similarly, probands of the remaining 77 pedigrees were initially analyzed using various targeted mutation or gene screening panels that were available in the past two decades [3] including *ABCA4* and recessive RP mutation panels (Asper biotechnology, Estonia), selected retinal disease gene resequencing arrays [140], targeted gene sequencing by Sanger sequencing and targeted exome capture [4] but failed to identify causative mutations.

#### Whole-genome sequence (WGS) Analysis

DNA isolation was performed using standard techniques from whole blood samples of patients using the Qiagen DNeasy blood kit (Qiagen, Germantown, MD) as previously described [7]. WGS was performed on at least one affected individual, and one or more unaffected close relative from each pedigree. The Illumina HiSeqX10 (Illumina, San Diego, CA) platform was used for sequencing whole-genomes at a minimum of 30X depth. The reads were mapped against human genome 19 (hg19) with decoy sequences using BWA-MEM [141, 142]. Biobambam2 was used to mark the duplicate reads and the remaining reads were sorted by genomic coordinate [143]. Variant calling was performed using HaplotypeCaller in Genome Analysis Toolkit (GATK) following the best-practice pipeline guidance [143, 144]. The genotyping quality of single nucleotide variants (SNVs) and insertions-deletions (INDELs) was assessed using the variant quality score recalibration approach implemented in GATK. Autosomal variants from pseudo-autosomal regions of the male X chromosome (chrX, 60001–2699520 and chrX, 154931044–155260560) were treated as diploid, whereas the rest of the male X chromosome, as well as the Y chromosome, were treated as haploid. A series of quality control processes were performed to determine the sample identity and sequencing quality, which includes sex identification based on the heterozygosity rate on the X chromosome, genetic relatedness among individuals was determined using identical by descent (IBD) segment analysis and this information was verified with the provided family structure, and sample heterozygosity rate was examined to detect any potential sample contamination. The sequencing data from five individuals from five different pedigrees were eliminated because they did not pass quality control metrics.

The called variants were annotated with SnpEff v4.11 [145], PolyPhen v2.2.2 [146], and CADD v1.3 [147]. Genome STRiP (svtoolkit 2.00.1611) [148] and Lumpy [149], which are part of the SpeedSeq software [150], were used to identify copy number variations (CNVs) in patients.

### ExAC Z score distribution in Retina genes

ExAC Browser (Beta) Exome Aggregation Consortium has a Z-score for each gene to evaluate its intolerance and conservation against three types of mutation; synonymous, missense and loss of function (LoF). The scores were originally developed to find disease-relevant de-novo mutations. In this study, we examined if the scores could be used to prioritize disease causative genes.

#### Filtering criteria

To identify rare deleterious SNVs, insertion-deletions (INDELs) and other types of structural variants as possible candidate variants, the following filtering criteria were used: allele frequency < 0.005 in 1000Genome project, < 0.05 in our inhouse cohort and < 0.05 in our 409 samples. Further, the allele frequency was validated using the ExAC and gnomAD databases. Highly deleterious variants were assessed and scored as: SnpEff putative impact = “HIGH” or PolyPhen2 Prediction = “possibly/probably damaging” or CADD Phred Score > = 30. Relatively deleterious variants were scored as: SnpEff putative impact = “HIGH/MODERATE” or Polyphen2 Prediction = “possibly/probably damaging” or CADD Phred Score > = 20.

Following initial filtering, selected variants were further analyzed based on segregation, pattern of inheritance, status reported in Human Genome Mutation Database (HGMD professional version 2020.4; http://www.hgmd.cf.ac.uk/ac/index.php), ClinVar classification on clinical relevance using the American College of Medical Genetics and Genomics (ACMG) and the Association for Molecular Pathology (AMP) guidelines, relevant population specific information and the known/reported physiological function of the corresponding gene [139].

#### Segregation analysis of SNVs

Segregation analysis of potentially disease-causing variant(s) identified in the IRD families by WGS was performed by dideoxy sequencing as previously described [151].

#### Segregation analysis of CNVs

Copy numbers variation of the exons of candidate genes and two reference genes *ZNF80* and *GPR15* were quantified using a CFX Connect Real-Time PCR Detection System (Bio-Rad Laboratories, Hercules, CA, USA) as described previously [11, 123].

#### Control sample analysis

A set of 95 unrelated ethnically matched Pakistani control samples were analyzed using dideoxy sequencing to validate novel variants identified in pedigrees from Pakistan, as described previously [8, 151]. A set of 768 individuals (including 422 whole-genome sequenced individuals from IRD pedigrees and 346 ethnicity matched controls) in our laboratory data set and 1000Genome data base and gnomAD database were used for the analysis of remaining variants.

#### qPCR analysis of structural changes

Segregation analysis of identified large insertion and deletions were validated by quantitative polymerase chain reaction (qPCR) analysis as described before [11].

## Supporting information

S1 Fig Clinical findings of patients with atypical genotypes. **(A) Fundus image from right eye of IV:2 from RF.VI13.0707 pedigree with the heterozygous *IMPDH1* c.940A>G, p.Lys314Glu variant.** The image demonstrates a waxy pallor of the optic disc, retinal vascular attenuation and mottling and light pigmentation of the retina in keeping with retinitis pigmentosa (RP). In addition, to normal RP features there is marked macular atrophy, with lacquer cracks are observed in the macular region of both eyes with clusters of pigment surrounding the area of atrophy. **(B to D) Fundus images of IV:1 and II:3 from pedigree RF.VI104.0514:** (F) Fundus images of IV:1 with the *CLN3* mutation show macula discoloration and early Bull’s eye pattern and subtle mottling of the retina at age 10. (G, H) Fundus images of II:3 show marked chorioretinal atrophy with large clusters of pigment suggestive of end-stage disease due to *C2orf71*. **(E) Composite left eye color fundus image of RF.VI111.0514 case, III:5 with mutations in *PRPF8* and *PRPF31* genes**. Image demonstrates way disc pallor, pigmentation of the fundus and retinal vessel attenuation suggestive of retinitis pigmentosa.
(TIF)

S1 Table Clinical diagnosis of all pedigrees. (XLSX)
