# Supplementary figures and images for: Deciphering the genetic architecture and ethnographic distribution of IRD in three ethnic populations by whole genome sequence analysis
Source: PLoS Genet. 2021 Oct 18;17(10):e1009848. doi: 10.1371/journal.pgen.1009848 (PMC8589175; doi:10.1371/journal.pgen.1009848)

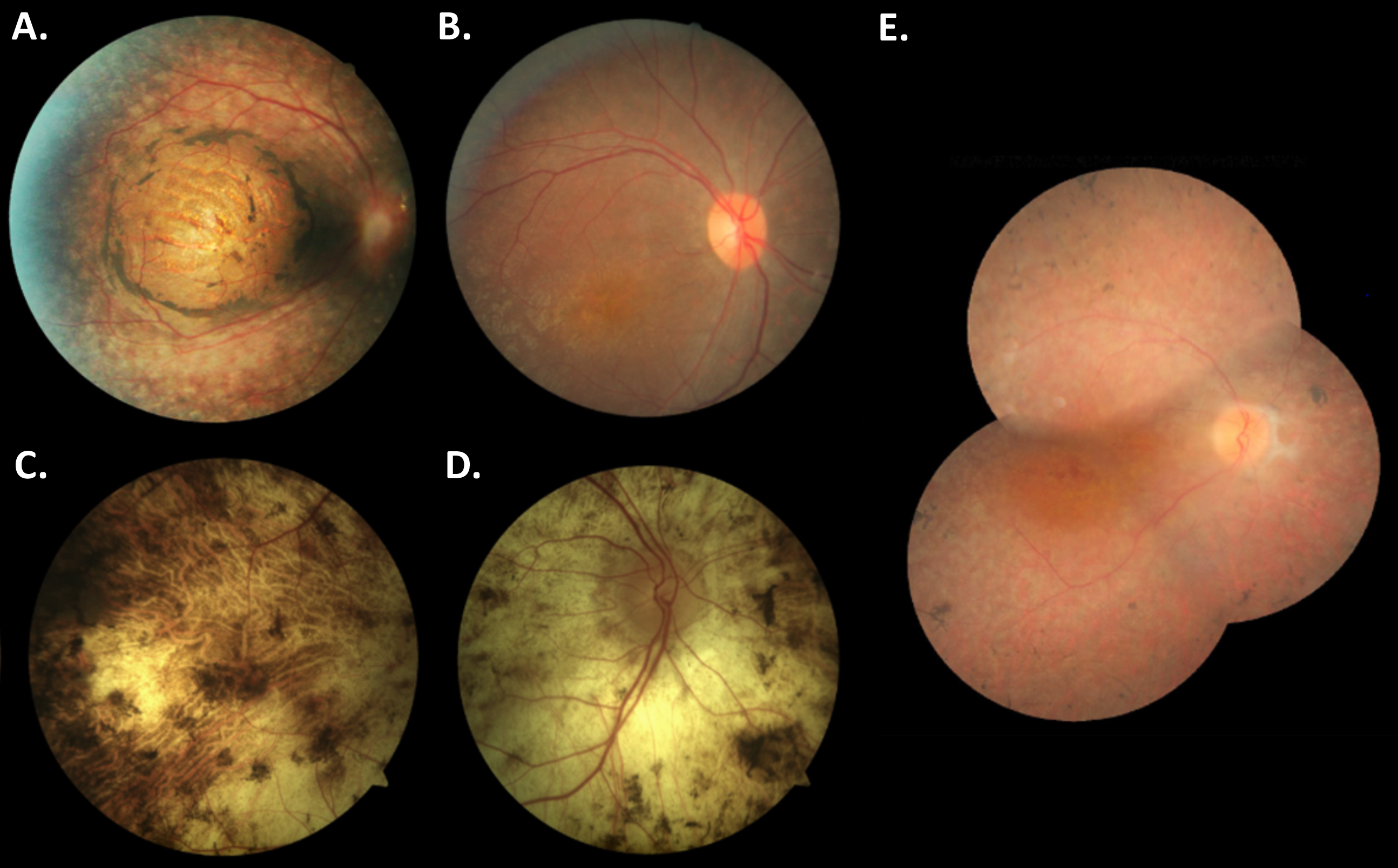

Supplement: S1 Fig — (A) Fundus image from right eye of IV:2 from RF.VI13.0707 pedigree with the heterozygous IMPDH1 c.940A>G, p.Lys314Glu variant. The image demonstrates a waxy pallor of the optic disc, retinal vascular attenuation and mottling and light pigmentation of the retina in keeping with retinitis pigmentosa (RP). In addition, to normal RP features there is marked macular atrophy, with lacquer cracks are observed in the macular region of both eyes with clusters of pigment surrounding the area of atrophy. (B to D) Fundus images of IV:1 and II:3 from pedigree RF.VI104.0514: (F) Fundus images of IV:1 with the CLN3 mutation show macula discoloration and early Bull’s eye pattern and subtle mottling of the retina at age 10. (G, H) Fundus images of II:3 show marked chorioretinal atrophy with large clusters of pigment suggestive of end-stage disease due to C2orf71. (E) Composite left eye color fundus image of RF.VI111.0514 case, III:5 with mutations in PRPF8 and PRPF31 genes. Image demonstrates way disc pallor, pigmentation of the fundus and retinal vessel attenuation suggestive of retinitis pigmentosa. (TIF) [file pgen.1009848.s001.tif]
